# Lignin-Based Mucus-Mimicking Antiviral Hydrogels with Enzyme Stability and Tunable Porosity

**DOI:** 10.1021/acsami.4c18519

**Published:** 2025-01-29

**Authors:** Sanjam Chandna, Tatyana L. Povolotsky, Chuanxiong Nie, Sophia Schwartz, Stefanie Wedepohl, Elisa Quaas, Kai Ludwig, Yulia Boyakova, Sumati Bhatia, Klas Meyer, Jana Falkenhagen, Rainer Haag, Stephan Block

**Affiliations:** a Institute for Chemistry and Biochemistry, Freie Universität Berlin, Berlin 14195, Germany; b Faculty of Science and Engineering, Department of Chemistry, Swansea University, Singleton Campus, Swansea, Swansea SA28PP, U.K.; c Federal Institute for Materials Research and Testing (Bundesanstalt für Materialforschung und -prüfung), Berlin 12489, Germany

**Keywords:** lignin functionalization, antivirals, mucus-mimicking hydrogels, tunable porosity, enzyme stability

## Abstract

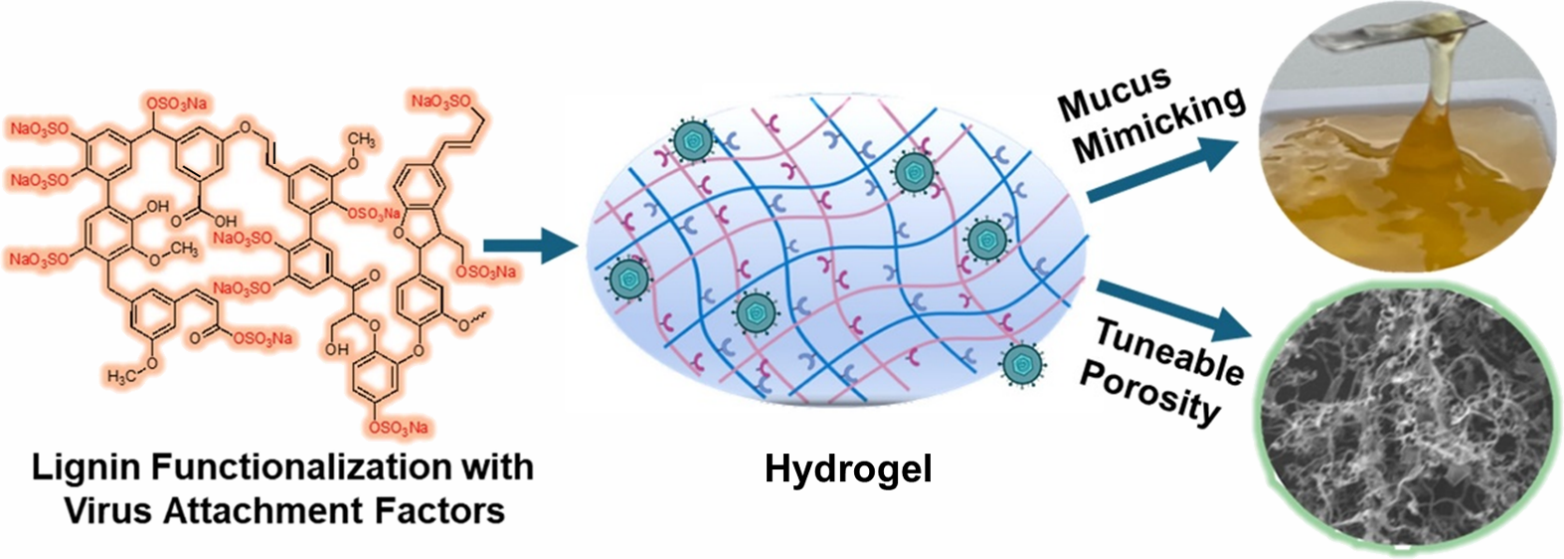

Mucus is a complex hydrogel that acts as a defensive and protective barrier in various parts of the human body. The rise in the level of viral infections has underscored the importance of advancing research into mucus-mimicking hydrogels for the efficient design of antiviral agents. Herein, we demonstrate the gram-scale synthesis of biocompatible, lignin-based virus-binding inhibitors that reduce waste and ensure long-term availability. The lignin-based inhibitors are equipped with sulfate moieties, which are known binding partners for many viruses, including SARS-CoV-2 and herpes viruses. In addition, cross-linking the synthesized inhibitors yielded hydrogels that mimicked native mucus concerning surface functionality and rheology. The degree of sulfation exhibits a very strong impact on the mesh size distribution of the hydrogels, which provides a new means to fine-tune the steric and electrostatic contributions of the virus–hydrogel interaction. This feature strongly impacts the sequestration capability of the lignin-based hydrogels, which is demonstrated by infection inhibition assays involving human herpes simplex virus 1, influenza A viruses, and the bacterium *Escherichia coli* (*E. coli*). These measurements showed a reduction in plaque-forming units (HSV-1) and colony-forming units (*E. coli*) by more than 4 orders of magnitude, indicating the potent inhibition by the lignin-based hydrogels.

## 1 Introduction

Hydrogels are three-dimensional networks of hydrophilic polymers that can absorb and retain large amounts of water or biological fluids while maintaining their structural integrity.^1−3^ Mucus hydrogels that line the biointerfaces play a vital role in both shielding the body from invading pathogens and facilitating the proper functioning of the underlying cells.^4^ Mucus forms the first line of defense for protecting epithelial cells by acting as a physical barrier for the passage of deleterious molecules.^5^ It allows the entry of nutrients and other vital molecules but protects our body from pathogens such as certain dangerous viruses and bacteria.^6^ The majority of mucus consists of water (∼95% w/w), and the rest is composed of mucins, globular proteins, salts, lipids, DNA, and cellular debris.^7^ Variable mesh size distribution is an important parameter for mucus hydrogels to bind the particles of interest, according to their size distribution.^8,9^ The mesh size of mucus hydrogels *in vivo* lies in the range of a few hundred nanometers, excluding larger objects such as bacteria and yeast cells.^7^ Objects less than the mesh size, such as viruses, are caught in the mucin network by sticking to the highly glycosylated structures in the mucosa and are removed from the body.^10^ Hence, along with acting as a barrier by affinity sorting, the mucus gels play the role of a filter.^11^ These properties inspired the development of mucin-mimicking macromolecules and mucus-mimicking hydrogels, which inhibit infections by forming barriers to pathogens, such as viruses.^12^

The performance of an antiviral strategy should not only be assessed based on its efficacy in inhibiting infections but also based on its sustainability. Therefore, to reduce the carbon footprint involved in the synthesis of such compounds, we aimed to employ a synthesis strategy that employs materials originating from natural sources and evaluated the possibility of forming mucin-mimicking macromolecules using lignin. Lignin comes up as an almost endless raw material produced through photosynthesis, which is currently significantly underutilized.^13−16^ As the second most abundant organic compound on earth, it is one of the main components of biomass that is thrown away in large quantities (approximately 70 million tons/year) as a byproduct of paper and pulp industries.^16−18^ It has an irregular 3D structure, which is mainly built from three subunits, i.e., guaiacyl (G), *p*-hydroxyphenyl (H), and syringyl (S).^1,19−24^ The complex organic structure of lignin provides this polymer with valuable properties, such as antioxidant, adhesiveness, UV-barrier, and antimicrobial properties.^25−28^ Owing to its highly diverse structure, good biocompatibility, and abundance in nature, lignin has the potential for biological applications and becomes a sustainable choice for the development of hydrogels in this work.^28−30^ Lignin and lignin-based hydrogels have been explored for different applications in literature due to the fascinating chemistry of lignin, decorated with numerous polyphenolic groups.^25,30−32^ An interesting avenue is the development of antiviral materials through the functionalization of lignin.

As many viruses carry a net charge on their surface (caused by the presence of specific proteins or glycoproteins),^33^ by modulating the hydrogel’s charge density, it is possible to create attractive or repulsive forces acting between virus and hydrogel, leading to virus binding or repulsion, respectively. For instance, herpes simplex virus type 1 (HSV-1), a pervasive pathogen responsible for oral and genital herpes, relies on host cell surface interactions mediated by heparan sulfates (a class of sulfated carbohydrates) during its initial stages of infection.^34−36^ It utilizes electrostatic interactions to bind with the heparan sulfates present on the cell surface to initiate infection.^36,37^ Heparan sulfates make up the outermost part of the cell membrane and the extracellular matrix.^35^ Therefore, the development of sulfated polymeric inhibitors with high binding affinity to herpes simplex virus is highly motivating for this work.

Recently, synthetic mucus biomaterials have been synthesized using four-arm polyethylene glycol (PEG) thiol and commercially available porcine gastric mucins (PGMs).^38^ These biomaterials can be applied to antimicrobial peptide delivery. This work attempts to design mucus-mimicking hydrogels using lignin as the starting material by modulating the charge, functionalities, rheological parameters, and porous network. The highlights of this work are (1) lignin functionalization with typical viral attachment factors, i.e., sulfate moieties; (2) hydrogel synthesis using sulfated lignin; (3) systematically tuning the mesh size and rheological properties of the hydrogels (designing hydrogels with mucus mimicking properties); and (4) testing the enzyme stability and sequestration efficiency of sulfated lignin hydrogels against HSV-1 and *E. coli* (to analyze the functionalized hydrogels for the broad-spectrum antimicrobial activity). This work aids in the development of highly sustainable and cost-effective infection control strategies to disrupt the interactions between pathogens and mucus. Such a synthetic mucus model can contribute to the advancement in mucus research by solving the problem of mucus recovery from animals and patients. Lignin-based mucin-mimicking hydrogels can also be potentially used to develop coatings for medical devices that prevent viral adhesion to mucosal surfaces.

## 2 Experimental Section

### 2.1 Sulfation of Lignin

For the sulfation of lignin (Sigma-Aldrich/Merck, cat. no. 370959), a sulfur trioxide pyridine complex was used. Lignin (400 mg) was mixed with sulfur trioxide pyridine complex (1.5 equiv w/w) in dry DMF (20 mL) at 60 °C for 20 h under an argon atmosphere. After the reaction time was over, the pH value was increased to pH 10 by the addition of sodium hydroxide solution (0.3 mol L^–1^). The product was dialyzed against sodium hydroxide solution (0.3 mol L^–1^), 10 wt % NaCl, and then water to remove the unreacted components.

### 2.2 Dynamic Light Scattering (DLS), Fourier Transform Infrared Spectroscopy (FTIR), and Elemental Analysis

For measuring the hydrodynamic diameter and surface charge (zeta potential), dynamic light scattering (DLS) was performed. The sample concentration of 0.3 mg mL^–1^ was prepared in deionized water and measured using the Zetasizer Nano series from Malvern Panalytical. All the samples were sonicated for 15 min before the measurement. Temperature equilibration was performed at 25 °C for 1 min. The measurements were performed for 10 scans of 15 s each, and the mentioned values for BL, SL1, and SL2 resulted from at least three measurements.

An Alpha II FTIR spectrometer from Bruker Optik GmbH with an ATR platinum diamond was used for FTIR analysis. Prior to FTIR analysis, the samples were completely dried using a lyophilization procedure. For liquid samples, a small droplet was applied directly to the ATR crystal. The conditions were 24 scans with a resolution of 4 cm^–1^. Data were collected across the spectral range of (e.g., 4000–600 cm^–1^).

The total carbon, hydrogen, nitrogen, and sulfur contents of the developed lignin hydrogels were determined by using a CHNS analyzer. For the elemental analysis, freeze-dried and crushed hydrogel samples (weighing 5–10 mg) were placed in a tin capsule and mixed with an oxidizer. The samples were then combusted at 1000 °C in a reactor. The resulting combustion products were analyzed by using a thermal conductivity detector set at 290 °C.

### 2.3 NMR Analysis

NMR experiments were performed on a 500 MHz NMR spectrometer system (VNMRS500, Varian Associates, Palo Alto, USA) operating at a proton frequency of 499.9 MHz (^31^P NMR at 202.4 MHz), which was equipped with a 5 mm OneNMR probe.

Gravimetry was performed on an ultramicrobalance (XP2 U/M, Mettler-Toledo, Gießen, Germany). Sample material, internal standard *N*-hydroxy-5-norbornene-2,3-dicarboxylic acid imide (e-HNDI), and relaxation agent Cr(acac)_3_ were accurately weighed and dissolved in the solvent mixture of CDCl_3_/pyridine 1:1.6 (v/v). Derivatization was performed as described in the literature by adding 100 μL of 2-chloro-4,4,5,5-tetramethyl-1,3,2-dioxaphospholane (TMDP) to the sample followed by shaking it for ∼1 h.^39^ Samples SL1 and SL2 show incomplete solubility; only the soluble phase was assessed by the NMR measurements for comparison. ^31^P NMR spectra on each sample were acquired by accumulating 256 scans at a pulse angle of 90° and a relaxation delay of 10 s.

### 2.4 Atomic Force Microscopy (AFM) Analysis

Samples for AFM imaging were prepared on mica coated with the cationic polymer poly(allylamine hydrochloride) (PAH). To this end, mica was washed with deionized water (ddH_2_O; Thermo Scientific Barnstead TKA-GenPure), incubated for 10 min with a 2 mM PAH solution (dissolved in ddH2O), and then washed again with ddH_2_O. The coated mica was then carefully dried with nitrogen gas flow over the surface and directly used for further sample preparation. Lignin samples were diluted to a final concentration of 0.01 mg/mL (0.05 mg/mL for bare lignin in ddH_2_O), and aggregates could only be observed undiluted with a concentration of 1 mg/mL. The lignin solution was incubated on the (coated) mica for 12–14 s before being washed off with ddH_2_O and then dried again with nitrogen gas.

Atomic force microscopy (AFM) images were obtained using a MultiMode Atomic Force Microscope (Veeco, Santa Barbara, CA) with a Nanoscope V controller and the NanoScope software. Imaging was performed with tapping mode in air using AC160TS cantilevers (Asylum Research by Oxford Instruments, LOT#753014). Experimental parameters of the images with a frame size of 1 × 1 μm were set to a scan rate of 1 Hz over 512 lines, an integral gain of 1, a proportional gain of 5, and a defined *z*-limit of also 1 μm. Images were taken of at least six different positions of each sample and analyzed using the MATLAB software.^40^

### 2.5 Gel Permeation Chromatography (GPC) and UV–Vis Spectrophotometric Analysis

A GPC system with the WinGPC UniChrom 8.2 software (PSS GmbH) was used for the GPC analyses. The system was operated with an isocratic pump (HPLC COMPACT PUMP 3350, Bischoff) at a flow rate of 0.5 mL/min. DMSO (HPLC grade) was used as eluent with a salt addition of 0.075 M NaNO_3_. A combination of four columns was used. At first, a precolumn (ABOA DMSO-Phil-P-250, 8 × 50 mm, 10^2^–7 × 10^4^ g/mol, AppliChrom) was used followed by two columns (DMSO-Phil-P-250, 8 × 300 mm, 10^2^–7 × 10^4^ g/mol, AppliChrom) and final separation column (DMSO-Phil-P-Multipore, 15 μm, 8 × 300 mm, 10^2^–10^6^ g/mol, AppliChrom). A column oven heated the separation columns to 60 °C (Thermostated Column Compartment). A refractive index detector (Shodex RI-501) and a photodiode array detector (SPD-M20A, Shimadzu Europe) were used to detect the fractions. The wavelength detector was operated at 280 nm. For calibration, 10 pullulan standards (PSS GmbH, now Agilent) were used. Samples of 2–3 mg/mL concentration were filtered through a 0.2 μm syringe filter before injection.

The UV-2600i UV–vis spectrophotometer from Shimadzu was used for absorbance measurements. The samples were diluted with deionized water to obtain a concentration of 0.3 mg/mL prior to analysis. All spectra were recorded over the wavelength range of 200–700 nm at room temperature. Quartz cuvettes with a 1 cm path length were used for the measurements. To quantify the amount of lignin present in different hydrogels, stock solutions of BL, SL1, and SL2 were prepared at a concentration of 1 mg/mL. These solutions were diluted in various concentrations to obtain the standard calibration curves of bare lignin (BL), SL1, and SL2. Then, the absorption of different hydrogels was measured at 280 nm as it corresponds to the absorption maxima of lignin. These values were used to find the concentrations of BL, SL1, and SL2 in the respective hydrogels after dialysis.

### 2.6 Cell Viability Test

Vero E6 cells (Leibnitz Institute DSMZ–German Collection of Microorganisms and Cell Cultures GmbH) were routinely cultured in DMEM supplemented with 10% FBS and 1% penicillin–streptomycin at 37 °C and 5% CO_2_. For the viability test, 100 μL per well of a 10,0000 cells/mL suspension in the medium was seeded into white 96-well plates and incubated overnight. The next day, the test compounds were solubilized to a concentration of 10 mg/mL in Milli-Q H_2_O or DMSO. Then, the compounds were further diluted in the cell medium as a 10-fold serial dilution, starting with the highest concentration of 1 mg/mL. For the sample solubilized in DMSO, a control containing only DMSO, diluted in the medium at the same percentages as used for the sample, was included. The supernatant of the cells was removed and replaced with 100 μL/well of the different compound dilutions in the medium. After 48 h of incubation, the supernatant was removed and replaced with 50 μL/well fresh medium and 50 μL/well CellTiter-Glo Reagent, which was prepared according to the instructions of the manufacturer (CellTiter-Glo Luminescent Cell Viability Assay, Promega). Well contents were mixed for 2 min on a shaker and incubated at room temperature for 30 min, and the luminescent signal was measured in a SPARK multimode microplate reader (Tecan). All tests were prepared three times independently in duplicates. Average values of the test wells were calculated, divided by the average values of untreated cells, and expressed as % viability ± standard deviation.

### 2.7 Synthesis of Lignin Hydrogels

Aqueous sulfated lignin solutions were prepared in various concentrations (0.5 to 2% w/v). This was followed by the addition of poly(acrylic acid) aqueous solution (2.8% w/v) to the reaction mixture and stirring at room temperature at a speed of 300 rpm for 5 min. Finally, ammonium persulfate (APS) (3% w/v) was added to the reaction mixture for the initiation of free radical generation, which mediated the cross-linking. The final volume of the reaction mixture was maintained to be 10 mL by the addition of deionized water as the solvent. The reaction vials were then transferred to an incubator and left undisturbed at 70 °C for 20 min. After being cooled to room temperature, the lignin-based hydrogels were dialyzed with PBS for ∼12 h using a 1 kDa membrane. This facilitates the removal of unreacted APS and other contaminants.

### 2.8 Rheology and Analysis of the Swelling Capacity of Hydrogels

The hydrogels were analyzed using a stress-controlled MCR 501 Anton Paar rheometer equipped with a stainless steel plate–plate geometry. All measurements employed a 25 mm diameter upper rotating plate with a fixed gap size of 0.15 mm. Testing was conducted at room temperature (25 °C), and each sample was allowed approximately 5 min to reach thermal equilibrium before measurement.

Strain amplitude sweep tests were conducted prior to the frequency sweep to determine the linear viscoelastic range (LVR), with strain amplitudes ranging from 0.01 to 100% at a constant frequency of 1.0 Hz. In the frequency sweep tests, a constant shear strain of 0.5% was applied, determined from preliminary tests to keep the material within the LVR. Measurements were taken across a frequency range of 0.1–10 Hz to thoroughly capture the hydrogels’ viscoelastic behavior.

Swelling capacity: For the analysis of the swelling capacity of hydrogels, the hydrogels were immersed in deionized water. At each time interval, swelling gels were withdrawn from the solution and weighed out after removal of the excess liquid from the gel surface. The excess water (or salt solution) was removed by filtration. The free swell water retention value (WRV), a measure of the dynamic water absorption properties of the gel, was calculated using the following eq 1:
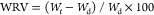
1where *W*_*t*_ is the weight of the wet gel at time *t* and *W*_d_ is the weight of the dried gel.

### 2.9 Enzyme Degradation Studies

The filtered enzyme solution was aliquoted and frozen at −20 °C. Then, 500 μL of hydrogels was pipetted into 2 mL Eppendorf tubes using cut tips (200 μL), also known as “wide bore”. This was followed by the addition of 100 μL of the enzyme solution/PBS to the tubes and thorough mixing by pipetting. The viscosity of the samples was measured directly after 24 h at 37 °C. The samples were temporarily stored at 37 °C without shaking.

### 2.10 Scanning Electron Microscopy (SEM)

The SEM analysis of the hydrogel samples was conducted using a Zeiss Sigma 300 VP field emission scanning electron microscope. The hydrogel samples were prepared 24 h prior to the measurements. For the internal matrix structure analysis of each hydrogel sample, the hydrogels were freeze-dried and then were sliced into thin sections using a blade. The freeze-dried sample was then adhered to a coverslip using a thin film coating of carbon and chromium. This coating served a dual purpose: it not only facilitated sample attachment but also minimized the buildup of static electric charge on the sample surface during electron irradiation, thereby reducing the risk of scanning artifacts and other imaging issues. Subsequently, the samples were subjected to gold coating (by spraying with colloidal gold particles) and allowed to dry at room temperature (25 °C) prior to analysis.

### 2.11 Virus Propagation and Titration

The virus was propagated on Vero E6 cells. Briefly, the Vero E6 cells were seeded in a T75 flask and were at >90% confluency for the infection. Then the cells were infected by HSV-1-GFP at MOI 0.1 for 2 days in DMEM (with 10% FBS and penicillin/streptomycin). Further, the cell culture supernatant was collected and centrifuged at 1000 rpm for 10 min. The supernatant after centrifugation was aliquoted and stored at −80 °C. The virus stock was titrated by a plaque assay on Vero E6 cells using MEM containing either 0.9% methylcellulose or 0.6% Avicel (90% microcrystalline cellulose/10% carboxymethycellulose) as the overlay. After being fixed, the plaques could be counted either under a fluorescent microscope or by eye after crystal violet staining.

### 2.12 *In Vitro* Antiviral Activity

#### Plaque Reduction Assay

The compounds were diluted 10-fold in 100 μL infection medium, and then 100 μL of approximately 4000 PFU/mL HSV-1-GFP dilution was added to the sample and incubated for 45 min at room temperature (r.t.). Then 100 μL of the virus was added onto Vero E6 cells and incubated at room temperature with moderate shaking for 45 min. Finally, the cells were washed with PBS once and then infected with the overlay medium for 2–4 days for plaque development. The plaques were counted as previously mentioned above.

#### Virus Binding Assay

The samples (5 μL each) were subjected to incubation with 100 μL of HSV-1-GFP solution (approximately 1 × 10^6^ PFU/mL) for 1 h. Then, the supernatant was collected and titrated by plaque assay on Vero E6 cells as described above. The binding with influenza A X31 virus was performed with the similar procedure except that the virus in the supernatant was titrated on MDCK II cells as reported earlier.^41^

### 2.13 Fluorescence Microscopy

After being fixed by 4% formaldehyde for 30 min, the cells were permeabilized with 0.1% Triton-X-100 in PBS at r.t. for 10 min followed by washing once with PBS. Afterward, DAPI (10 μg/mL) was added to stain the cell nuclei for 10 min. The cells were then washed with PBS, and the images were acquired with a fluorescence microscope (Axio Z1, Zeiss, Germany).

### 2.14 *In Vitro* Antibacterial Activity via Growth Curves and CFU Assays

The K-12 derivative *E. coli* AR3110 was streaked out on LB agar from frozen stock. A single colony was used to create the liquid culture that was grown to an OD_600_ of 0.600–0.800 at 37 °C in LB media with shaking. The culture was then diluted in LB to a final OD_600_ concentration of 0.01, and 100 μL of this culture was added to 100 μL of UV-sterilized hydrogel of varying concentrations in a 96-well plate (round bottom, Sarstedt). Each well was thoroughly mixed by pipetting. The lid of the 96-well plate was coated in antifog solution (0.05% Triton X-100, 20% ethanol in water) and allowed to completely dry under the clean bench hood. The 96-well plate (closed with a lid) was placed in an Agilent BioTek Epoch 2 Microplate Spectrophotometer, incubated for 20 h at 37 °C with continuous shaking (double orbital), and OD_600_ was measured in 15 min intervals. The OD_600_ after 20 h was used for graph generation. Three biological replicates were performed each consisting of three technical replicates. Error bars represent ± SD. Statistical analysis was executed using the GraphPad Prism software.

Colony forming units (CFUs) were then calculated after the 20 h growth curve using the resultant culture as described in Maan et al.^42^ Briefly, the culture was transferred to a 96-well plate, and a serial dilution from 10^0^ to 10^–7^ was performed in Dulbecco’s Phosphate-Buffered Saline (DPBS). From each dilution, 20 μL was transferred onto LB agar plates and allowed to dry completely in a clean bench hood. Plates were incubated overnight at 37 °C, and colonies were then counted. The colony forming units (CFUs) were calculated using the eq 2:
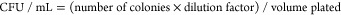
2Three biological replicates were performed each consisting of three technical replicates. Error bars represent ± SD. Statistical analysis was executed using the GraphPad Prism software.

## 3 Results and Discussion

### 3.1 Concept of the Sulfated Lignin and Hydrogel Synthesis

**Scheme 1:**
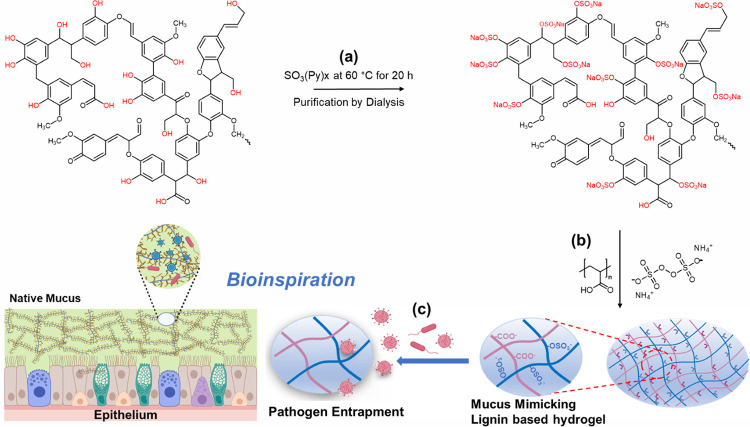
(a) The Sulfation Process of Lignin, (b) Subsequent Synthesis of Lignin-Based Hydrogels by Free Radical Polymerization at 70°C, and (c) Pathogen Entrapping Studies of the Hydrogel, Which Is Designed to Mimic Mucus (Inspired by Native Mucus)

**Figure 1:**
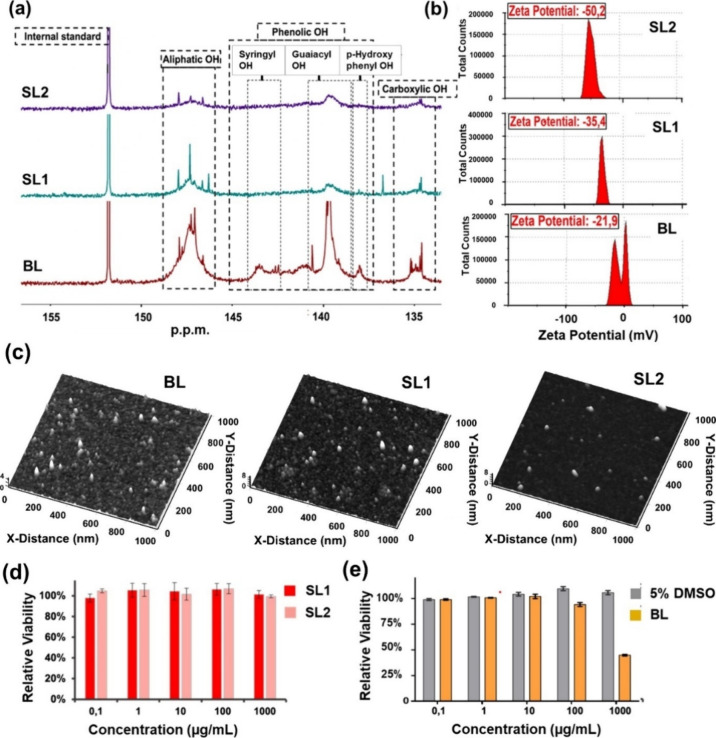
(a) ^31^P NMR analysis of the bare and sulfated lignin. (b) Analysis of surface charge through zeta potential analysis of bare lignin (BL) as well as the sulfated lignins SL1 and SL2. (c) AFM images obtained using tapping mode in air for bare lignin (BL), medium sulfated lignin (SL1), and sulfated lignin (SL2), with an average monomeric particle size of 0.63 nm. (d, e) Relative viability of Vero E6 cells after 48 h of exposure to the compounds as indicated in the legend.

Mucus is a complex aqueous fluid that is made up of smaller proteins, lipids, electrolytes, and the glycoprotein mucin.^4^ These components give mucus its viscoelastic, lubricating, and hydrating qualities. Owing to the presence of sulfate groups and sialic acid residues on glycoproteins, mucus carries substantial negative charges. Such residues typically act as pathogen attachment factors and help in the exclusion of infectious agents from the body through the mucosa linings present in various parts of the body. This inspired us to functionalize lignin with sulfate groups (−OSO_3_^–^) through sulfation of hydroxyl groups.^14^ The functionalization strategy of kraft lignin with sulfates to generate the sulfated lignin is represented by the chemical scheme in Scheme 1. Since the yield of the sulfation reaction of lignin is typically ∼95%, the reaction is highly scalable (up to several grams). After sulfation, the product was subjected to dialysis for a week to remove the unreacted components. The success of the functionalization was confirmed by ^31^P NMR (Figure 1; Table S1, Supporting Information) and elemental analysis (Figure S1b,c, Supporting Information). For both lignin and lignin-based hydrogels, the carbon content (%C) decreases from BL to SL2, indicating progressive structural changes. The hydrogen content (%H) increases in hydrogels as compared to non-cross-linked lignin due to water incorporation and enhanced hydrogen bonding. The sulfur content (%S) significantly increases in both SL2 and SL2-H, which confirms the successful incorporation of sulfur moieties during lignin modification and their retention in hydrogel structures.

Interestingly, after sulfation, lignin was found to be completely soluble in water, enabling the generation of lignin-based mucus mimicking hydrogels with high binding capacity for viruses. Previously, our group has developed polyglycerol-based mucus-inspired hydrogels, synthesized through thiol oxidation to form disulfide bonds between the cross-linker and the linear precursors.^12^ In another such attempt to synthesize mucus-inspired hydrogels using polyglycerols by our group, highly promising antiviral activity was obtained against HSV-1 and respiratory syncytial virus (RSV).^43^ In this work, we attempted to utilize a highly abundant and sustainable material, i.e., lignin, for the functionalization process and development of bioinspired mucus mimicking hydrogels for the first time. For the synthesis of sulfated lignin-based hydrogels, the sulfated lignin was copolymerized with poly(acrylic acid) (PAA) using ammonium persulfate (APS) as a free radical initiator; this is described in Scheme 1. The structure of lignin is based on the structural model of kraft lignin by Adler.^13^ The reaction mixture containing lignin, poly(acrylic acid) (PAA), and ammonium persulfate (APS) was heated to 70 °C with deionized water as a solvent. Upon heating, APS decomposes to generate free sulfate free radicals. The thermal energy also disrupts the hydrogen bonding between the PAA chains and between PAA and water molecules. The sulfate radicals (generated from heating APS) abstract hydrogen atoms from the PAA backbone, creating radicals on the polymer chain. Similar effects can also happen with the lignin polymer, and therefore, radicals are generated on lignin’s phenolic/aliphatic hydroxyl as well as carboxylic groups. This leads to intra- and intermolecular cross-linking reactions of radicals, resulting in the generation of a stable lignin-PAA grafted copolymer as a hydrogel (Scheme 1 and Figure 1a).

The synthesis process was straightforward and simple, carried out in 30 min. The use of PAA in this work, rather than acrylic acid (which is more commonly used), eliminates the need for an acrylamide cross-linker. A pure PAA-based hydrogel was also synthesized as a control by cross-linking PAA using APS with a similar amount in an aqueous environment (Figure S10a, Supporting Information). The PAA hydrogel was synthesized in a similar time; however, it has a very soft consistency as compared to the lignin-PAA hydrogel. After dialysis, the retained lignin concentrations for BL-H, SL1-H, and SL2-H are 89.1, 94.8, and 95.1%, respectively. This indicates that the unbound bare lignin is approximately 11%, which is removed during the dialysis process. This value reduces to approximately 5% for sulfated lignin (SL1 and SL2). The functionalized lignin powder and hydrogels were then subjected to characterization.

### 3.2 Physical Characterization of the Functionalized Lignin Powders

To assess the impact of the degree of sulfation (DoS) on the physicochemical properties of lignin powder, we aimed for sulfated lignin (denoted as SL1 and SL2 in the following), in which approximately 78% (SL1) or 90% (SL2) of the available hydroxyl groups were converted into sulfates. This conversion was monitored based on the ^31^P NMR chemical shifts in the region of 146 to 148 ppm, which correspond to aliphatic hydroxyl groups and show a significant decrease in the cases of SL1 and SL2 (Figure 1a).^44^ Moreover, chemical shifts were observed in the region of 137 to 145 ppm corresponding to the phenolic groups in lignin (syringyl, guaiacyl, and *p*-hydroxyphenyl).^44^ The amounts of aliphatic hydroxyl and phenolic units were calculated in the bare lignin (BL), SL1, and SL2 (summarized in Table S1 of the Supporting Information).^45^ The decrease in these groups is paralleled by an increase in sulfate groups as indicated by elemental analysis (Figure S1b, Supporting Information), which proves the successful sulfation of lignin. Under the assumption that the decrease of hydroxyl and phenolic units (quantified by ^31^P NMR) is caused by the full conversion to sulfate moieties, degree of sulfation (DoS) values of 78% (SL1) and 90% (SL2) are obtained for sulfated lignin (Table 1). Similar DoS values are obtained from the results of the elemental analysis (i.e., after correlating with the maximum weight percentage of sulfur that can be incorporated in each subunit of lignin) (Figures S1 and S2, Supporting Information).

**Table 1:** Degree of Sulfation, Hydrodynamic Radius, PDI, Zeta Potential, SEC-Determined Molecular Weight, and IC_50_ Values for the Interaction of the Lignin with Human Herpes Simplex Virus 1 (HSV-1)

**sample**	**degree of sulfation**	**particle size (nm)**	**polydispersity index (PDI)**	**zeta potential (mV)**	**molecular weight, Mw** (g/mol)	**IC**_**50**_**values (nM)**
BL		309 ± 16	0.246	–21.9 ± 2.6	7278.6	11.9 × 10^3^
SL1	78%	216 ± 9.8	0.460	–35.4 ± 1.7	9535.5	302.5
SL2	90%	167 ± 8	0.339	–50.2 ± 3.2	8641.3	57.8

The surface charge of the lignin samples was measured through zeta potential analysis (Figure 1b), which is a measure of the electrostatic charge at the surface of particles or molecules in a colloidal dispersion.^46,47^ There were two peaks observed in the case of bare lignin (BL), which highlight the presence of different particle populations in raw lignin with varying surface charges. While lignin does contain charged functional groups, such as phenolic hydroxyl groups and carboxylic acids, a significant portion of its structure consists of uncharged molecules, primarily carbon–carbon linkages. The addition of sulfate groups to lignin significantly alters its zeta potential. Therefore, the values of zeta potential shift toward more a negative side, i.e. from −21.9 mV in the case of bare lignin to −35.4 mV (SL1) and −50.2 mV (SL2), respectively (Figure 1a). This increase in the negative charge of lignin further is in line with the results from ^31^P NMR and elemental analysis, which indicate the incorporation of negatively charged sulfates into the lignin.

For the determination of the molecular weight distribution of bare and sulfated lignin, size exclusion chromatography (SEC) was performed (Figure S1a, Supporting Information). The molecular weight (*M*_w_) of the bare (unmodified) lignin was deduced to be 7300 g/mol. It was observed that the peak in the chromatogram becomes narrower and the *M*_w_ (weight-average molar mass) increases upon sulfation to 9540 (SL1) (∼1.3×) and 8650 g/mol (SL2) (∼1.2×), respectively. This clearly indicates that the molecular weight distribution of sulfated lignin shifts to larger values and becomes more monodisperse (Figure S1a and Supporting Information). Such molecular weights of the lignin particles indicate their size range in the nanometer scale, which was confirmed by atomic force microscopy (AFM) analysis of the bare and sulfated lignin samples (Figure 1c and Figure S3, Supporting Information).^48^ For the bare lignin, a macromolecule of approximately 10 kDa, a size on the order of 1 nm was expected, and experimental parameters were set accordingly.^53^ All lignin samples were found to consist of predominantly monomeric particles with a size of 0.63 nm, as shown in Figure 1c and Figure S3 and Supporting Information. SL1 and SL2 exhibit a very sharp peak at the value of 0.627 nm. There is no measurable change in monomeric particle size after the sulfation of lignin. The broader distribution of bare lignin particles here is attributed to a superposition of two peaks: the monomeric complex at around 0.63 nm and a dimeric complex at 0.84 nm. Besides monomeric particles, large aggregates were observed at higher concentrations of the bare lignin. For bare lignin, the existence of a nanometer-sized species was confirmed by the particle size distribution analysis using dynamic light scattering (DLS; Figure S4, Supporting Information). Nevertheless, as the scattering intensity shows a very strong dependence of the molecular weight in this size regime, the DLS size distribution strongly overestimates the occurrence of the large complexes with respect to nm-size species. Hence, both methods, DLS and AFM, yield complementary results that have to be taken into consideration when determining the particle size distribution of complex samples. The values for the size distribution, polydispersity index (PDI), zeta potential, and molecular weight analysis are summarized in Table 1. In addition, UV–visible spectroscopy was conducted with bare and sulfated lignin samples. For comparison between the bare and sulfated lignin samples, the spectra were also normalized at 450 nm. It was observed that the absorption peak was ∼280 nm for bare lignin and ∼276 nm for sulfated lignin (SL2). A small low energy peak was also observed at ∼360 nm for bare lignin, which can be attributed to conjugated structures such as enol ethers (Figure S5, Supporting Information).^23^

To gain further insights into the impact of sulfation on the chemical structure of lignin, FTIR spectra of sulfated lignin and bare (unmodified) lignin were determined (Figure S6 and Supporting Information). A sharp peak was observed between the region of 1200 and 1300 cm^–1^, which is, in agreement with previous studies, attributed to sulfate groups (−SO_3_H).^20,49^ There were also peak shifts observed in the CO stretching of lignin from ∼1020 to ∼1100 cm^–1^. Moreover, a sharpening of the OH peak at ∼3500 cm^–1^ was observed indicating intramolecular OH bonding.^19,20,50^ As mucus is a natural substance in the body, polymers designed to mimic mucus should be biocompatible and safe for use in biological systems without causing harm or triggering an immune response. To investigate the potential of the sulfated lignin polymers, the viability of VeroE6 cells (as a model for fibroblast cells, which are also used for virus propagation) was tested after 48 h of exposure (Figure 1c).

It was observed that cells exposed to SL1 or SL2 showed no reduction in their viability up to a polymer concentration of 1 mg/mL, indicating high compatibility with Vero E6 cells. The bare lignin showed a decrease in the cell viability at the concentration of 1 mg/mL, which emphasizes that bare lignin is comparatively more toxic to the Vero E6 cell lines than the sulfated lignin (Figure 1c). The addition of DMSO was not necessary for the sulfated lignin samples as they were completely soluble in water. Interestingly, the biocompatibility of the sulfated lignin hydrogels increased significantly as compared to that of the bare lignin. Therefore, it can be concluded that by introducing sulfate moieties onto lignin’s intricate macromolecular architecture, the resulting derivatives (1) exhibit improved solubility in aqueous solution and (2) increase the biocompatibility of the lignin.

### 3.3 Hydrogel Characterization: Rheology, Enzymatic Stability, and Electron Microscopy

**Figure 2:**
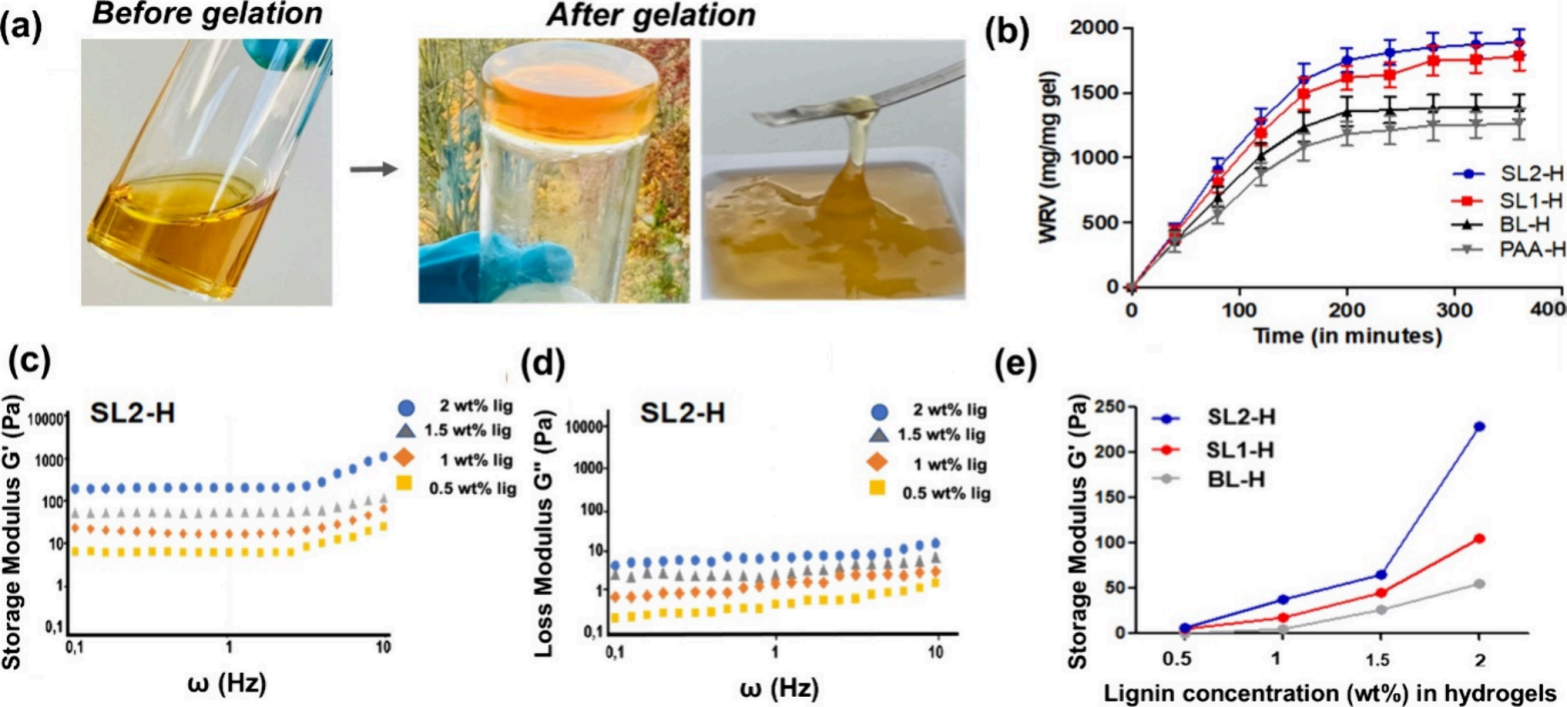
(a) Pictorial representation of the synthesized hydrogel before and after gelation. (b) Swelling capacity analysis of the bare (BL-H), sulfated lignin hydrogels (SL1-H, SL2-H), and PAA hydrogel (PAA-H). (c) Storage modulus of sulfated lignin (SL2-H) at different concentrations. (d) Loss modulus of sulfated lignin (SL2-H) at different concentrations. (e) Effect of the degree of sulfation on the storage modulus of hydrogels.

After the detailed analysis of the surface characteristics, the bare and sulfated lignin powders were subjected to hydrogel synthesis. Figure 2 describes the visual appearance, swelling capacity, and rheology of the bare (BL-H) as well as sulfated lignin hydrogels (SL1-H and SL2-H). The parameters for the hydrogel synthesis were designed and optimized carefully to mimic the native mucus hydrogels. The high solubility of the sulfated lignin in an aqueous medium facilitated the hydrogel synthesis through a sustainable and mild process. Therefore, the functionalized lignin hydrogels were synthesized using a free radical polymerization approach, as indicated earlier in Scheme 1. The excess of small sulfated residues and ammonium ions is removed during the dialysis process after the gelation. The FTIR analysis of the lignin hydrogels reveals the presence of a C=O stretch at ∼1697 cm^–1^, C–C–O stretch at 1164 cm^–1^, and O–C–C stretch at 1044 cm^–1^ (Figure S10b, Supporting Information).^50^ There is also possibility of C–O (ether) or C–C single bond formation due to intramolecular cross-linking in lignin, and this is indicated by the FTIR peaks at 1151 cm^–1^ (C–O stretching) and 1419 cm^–1^ (aromatic C–C stretching) (Figure S10b). The single sharp peak at 1697 cm^–1^ in SL2-H (sulfated lignin-PAA hydrogel) can be attributed to the carboxylate groups coming from PAA.

To analyze the structural differences between the PAA homopolymer and PAA hydrogel, we performed an FTIR (ATR) analysis of these samples (Figure S10a). It was observed that there is a single peak at 1605 cm^–1^ in the PAA hydrogel instead of two peaks at 1699 and 1610 cm^–1^ in PAA. As these peaks indicate the C=O stretching of carboxylic acid (−COOH) groups, a single peak at 1605 cm^–1^ indicates an increase in carboxylate ion (−COO^–^) formation and also asymmetric stretching of carboxylate ions (−COO^–^). Moreover, cross-linking significantly alters the molecular structure and the environment around the carboxylic groups. Another striking observation is the shift of the peaks in the region of 1100–1300 cm^–1^ toward higher wavenumbers in the PAA hydrogel. This indicates altered vibrational modes due to hydration and formation of carboxylate ions. The deepening of the broad peak between 3200 and 3400 cm^–1^ is observed due to enhanced hydrogen bonding and structural changes due to cross-linking within the hydrogel.

As hydrogels are polymeric materials that can swell in water and retain a significant fraction of water within their structure, the swelling rate is one of the most important properties of hydrogels.^51^ To measure the swelling rate of sulfated lignin and bare lignin hydrogels, the profile of swelling capacity versus time of the hydrogel samples was obtained by performing free-absorbency capacity measurements at consecutive time intervals. First, the synthesized hydrogels were subjected to freeze-drying, and then, the completely dried hydrogels were kept inside water for swelling. It was deduced that the free swell water retention value (WRV) of the sulfated lignin hydrogel was approximately 1600, which was reduced to ∼1454 in the case of bare lignin hydrogels (Figure 2b). The swelling capacity of the synthesized hydrogels (BL-H, SL1-H, and SL2-H) was compared to that of the poly(acrylic acid) (PAA) hydrogels (PAA-H), and it was found to be significantly high. Therefore, it can be concluded that all of the hydrogels exhibit high water absorption abilities. Further details regarding the swelling capacity analysis are also mentioned in the Supporting Information, Figure S11.

The mechanical properties of a substance are indicated by its viscoelastic properties, which can be evaluated through oscillatory rheology experiments. Mucus hydrogels are viscoelastic and sticky, effectively trapping and immobilizing foreign particles and pathogens. Viruses, bacteria, and other microorganisms can become entangled in the mucus matrix, preventing them from reaching and infecting the underlying cells. For a hydrogel to mimic native mucus, its storage modulus and loss modulus should lie in the range of 1–10 and 0.1–1 Pa, respectively.^54−56^ The strain amplitude sweep tests were performed prior to the frequency sweep to determine the linear viscoelastic range (LVR), represented in Figure S7 (Supporting Information) (as a function of strain amplitude from 0.01 to 100% at a constant frequency of 1.0 Hz). During the frequency sweep tests (Figure 2c–e), a constant shear strain of 0.5% was applied based on preliminary tests to ensure that the material remained within the LVR. Measurements were conducted over a frequency range of 0.1–10 Hz to capture the viscoelastic behavior of the hydrogels comprehensively. All the experiments were conducted at 25 °C. The rheological behavior of hydrogels formed using bare lignin (BL-H) or sulfated lignin (SL1-H and SL2-H) is shown in Figure 2c–e, and it was speculated that all the synthesized hydrogels exhibited a significant elastic-dominant behavior, therefore proving the stability of the cross-links. The concentration of lignin during hydrogel formation was optimized to modulate the rheological properties of the developed hydrogel. It was observed that the hydrogels with a lignin concentration of 0.5 wt % exhibited the desired elastic and viscous modulus values resembling the native mucus.

Spinnability in hydrogels is their capability of thread formation, arising from non-Newtonian flow.^54^ For this, an important prerequisite is the existence of both viscous and elastic properties.

Initially, at a lignin concentration of 2 wt %, the hydrogel obtained was rigid and exhibited high viscoelastic properties, and it also did not tend to flow within the measured time period. It is suggested from the results that the synthesized lignin hydrogel is also quite elastic as it shows minimal flow upon pressure application, and as the pressure is removed, it recoils quickly back to its previous position. However, as the hydrogel concentration decreases from 2 to 0.5 wt % of lignin, it appears to be a viscous hydrogel with high spinnability, maintaining its network structure and elasticity.

Varying the concentration of lignin between 0.5 and 2% revealed a significant impact on the storage and loss modulus (*G*′ and *G*″, respectively) of the resulting hydrogel (Figure 2c–e, Table 2). It was observed that upon sulfation of the lignin hydrogels, the storage modulus increased by roughly 2 orders of magnitude (Figure 2e). Figure 2c–e represents the rheology data of SL2-H, and the data of SL1-H and BL-H can be found in Figure S8a (Supporting Information). This finding was used to optimize the synthesis parameters to achieve the *G*′ and *G*″ values of the order of native mucus. The rheology of PAA hydrogels was recorded as control (Supporting Information, Figure S8b); it was found that the storage modulus and loss modulus of PAA hydrogels were ∼450 and ∼20 Pa, respectively.

**Table 2:** Storage (*G*′) and Loss (*G*″) Modulus Values of Lignin Hydrogels as a Function of Radial Frequency (*w*) of 1 Hz at 37 °C

**lignin concentration(weight %)**	**storage modulus (Pa) (***G***′)**			**loss modulus (Pa) (***G***″)**		
	BL-H	SL1-H	SL2-H	BL-H	SL1-H	SL2-H
2	55.56	105.6	229.1	6.6	5.5	6.8
1.5	26.63	45	64.9	2.14	2.2	2.4
1	5.82	17.3	37.8	0.6	1.9	1.52
0.5	0.13	4.9	6.7	0.03	0.8	0.48

**Figure 3:**
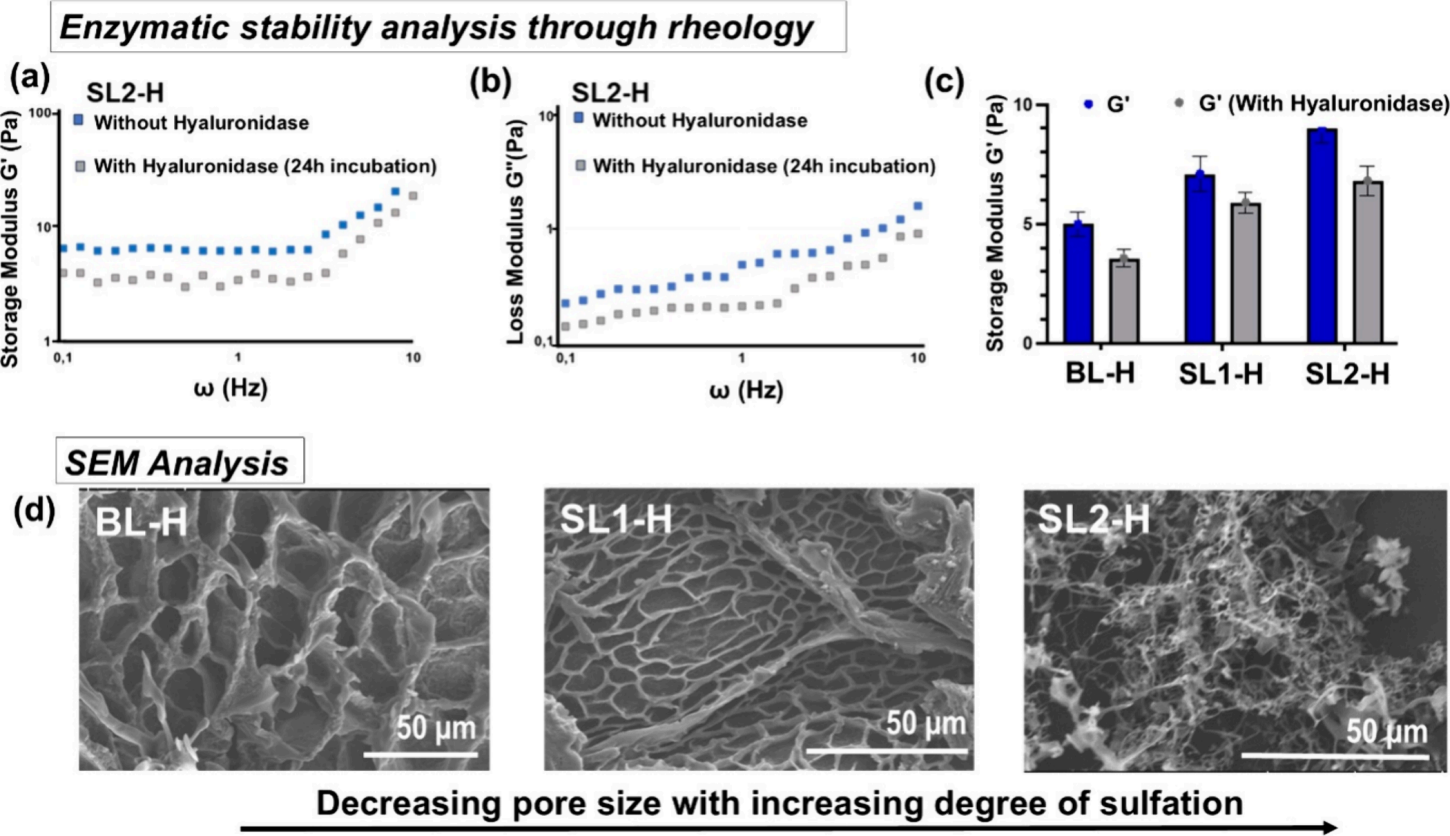
(a) Effect of enzyme (hyaluronidase) incubation on the storage modulus of hydrogels. (b) Loss modulus plots of SL2-H after incubation with hyaluronidase. (c) Comparison of Storage moduli of BL-H, SL1-H, and SL2-H after incubation with hyaluronidase. (d) SEM analysis of bare lignin hydrogel (BL-H) and sulfated lignin hydrogels (SL1-H and SL2-H).

The synthesized hydrogels were tested for their stability against enzymatic cleavage by the enzyme hyaluronidase and their porous structure (Figure 3). Hyaluronidase is an enzyme present in synovial fluid, which is responsible for the lubrication of joints and acts as a shock absorber. It helps reduce friction between bones and cartilage during joint movement, contributing to joint health and mobility. To test the stability of the synthesized hydrogel in the presence of hyaluronidase, hydrogels were incubated with the enzyme at 37 °C for 30 h followed by evaluating the hydrogel rheology and measuring changes of the storage and loss moduli. It was observed that the viscosity of all the hydrogels (BL-H, SL1-H, and SL2-H) was affected by hyaluronidase incubation; however, the hydrogels still maintained storage modulus values between 1 and 10 Pa until the frequency of 5 Hz (Figure 3a,b). Similarly, after enzyme treatment, the loss moduli of the BL-H, SL1-H, and SL2-H were in the range of 0.1 to 1 Pa, which highly resembles native mucus (Figure 3c). Further incubation of the hydrogels with hyaluronidase did not affect their rheology and physical structure or appearance. This analysis highlights the stability of the lignin-based hydrogels against enzymatic degradation by hyaluronidase.

The structure and composition of mucus play an important role in maintaining barrier properties by acting as a filter for the diffusion of biomolecules and pathogens. To gain insights into the surface morphology and internal structure of the hydrogel, the lignin hydrogels were subjected to scanning electron microscopy (SEM) analysis, which provided valuable insights into the surface morphology and partially into the internal structure of the hydrogel (Figure 3d). In the SEM images of SL1-H and SL2-H (1 w/v % lignin), the surface of the sulfated lignin hydrogels appeared to be highly porous (Figure 3d and Figure S9a, Supporting Information), with morphological features on the length scale of 1 μm for SL2-H, 10 μm for SL1-H, and significantly exceeding 10 μm for BL-H. Such observations resemble well with the native mucus, which was a significant and motivating finding.^6,57^ Furthermore, the SEM images indicated that increasing the DoS caused the mesh size distribution to shift toward smaller values, which is in line with the observed increase in (shear) storage modulus *G*′ upon sulfation (see Figures 2e and 3c). The plateau value of *G*′ is known to depend of the mesh size ξ and the stiffness of the cross-linker(s) (describable, e.g., in terms of the cross-linker’s persistence length *l*_p_) and to scale with the ratio , which indicates that *G*′ increases if the persistence length *l*_p_ increases and/or if the mesh size ξ decreases.^52,53^ As the latter has a much larger exponent, *G*′ will be much more susceptible to changes of the mesh size than the persistence length. In fact, SEM imaging indicated that sulfation induced a change in hydrogel structure from coarse network of thick fibers (with persistence length *l*_p_ and mesh size ξ ≫ 1 μm; Figure 3d, left) to a fine network of thin fibers (with persistence length and mesh size ≪ 1 μm; Figure 3d, right), being perfectly in line with the observation of an increase of *G*′ with the increasing level of sulfation.

This observation was made at different weight concentrations of the hydrogel, which pointed toward the interesting possibility of tuning the mesh size distribution of lignin hydrogels using the appropriate degree of functionalization, i.e., sulfation. There are few reports on tuning the mesh size distribution of hydrogels using variable concentrations of the precursor;^58^ however, the effect of the degree of functionalization of the precursor molecule on the mesh size of hydrogels is still an unexplored area. This interesting finding can be utilized in different fields to tune the porosity of the hydrogels by varying the degree of functionalization of the precursor molecule. The SEM images of the PAA hydrogel were recorded as control, which revealed the good porosity of such hydrogels (Figure S9b, Supporting Information). This finding is in line with the high swelling capacity of pure PAA hydrogels as mentioned previously.

### 3.4 Pathogen Binding

**Figure 4:**
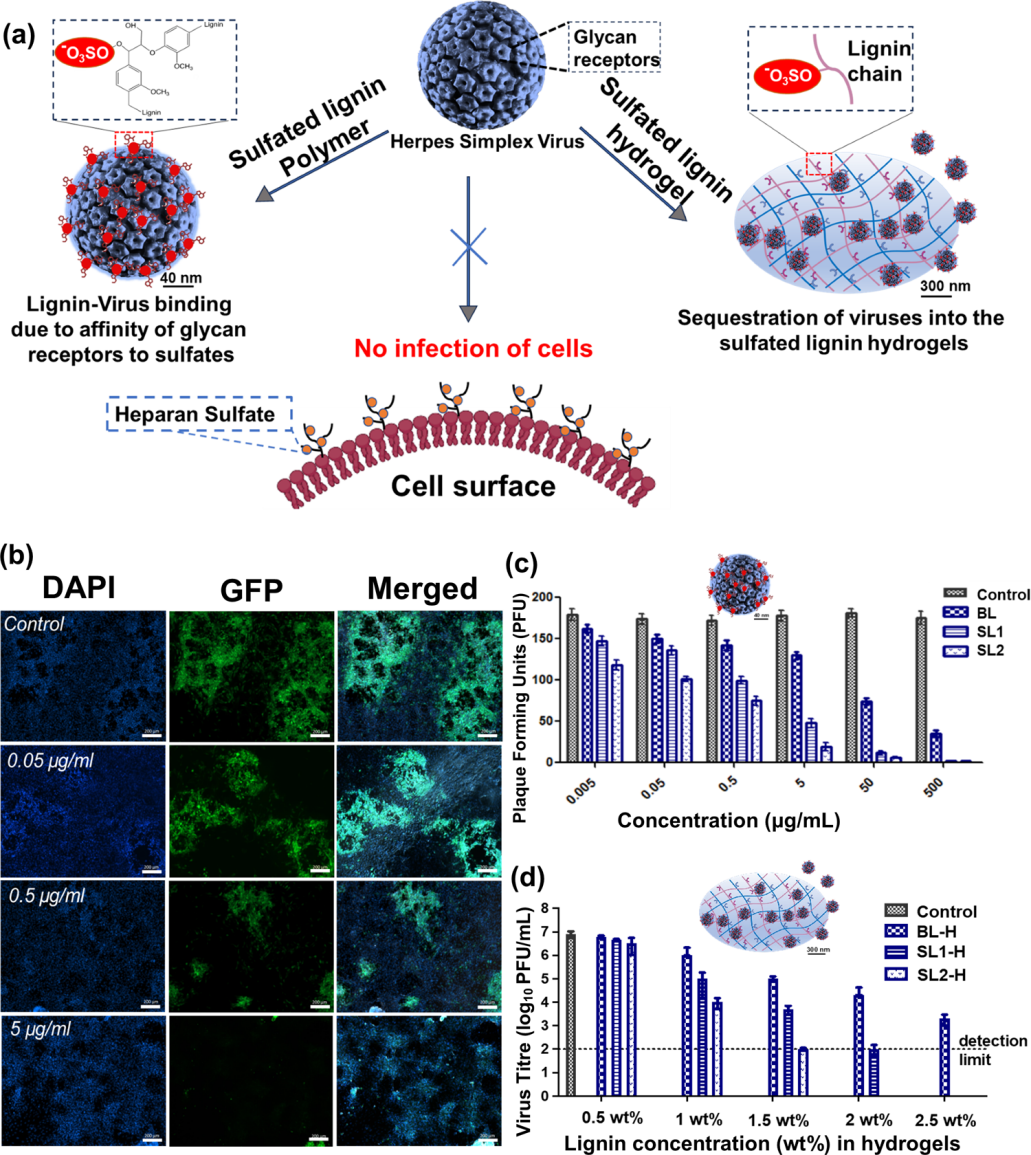
(a) Concept of HSV-1 inhibition through sulfated lignin powder and hydrogel. (b) Initial examination of the HSV-1 inhibition through fluorescence microscopy at different concentrations of sulfated lignin (scale 200 μm). (c) Quantitative estimation of HSV-1 inhibition by lignin powders. (d) Quantitative estimation of HSV-1 inhibition by lignin hydrogels with different weight percentages of lignin.

Inhibiting virus entry into host cells at an early stage of infection is a promising concept for the prevention and treatment of infections. HSV-1 binds to the heparan sulfates on the host cell surface via its surface proteins gB and gC.^12,59^ It can be hypothesized that the presence of lignin (functionalized with sulfate moieties) competitively inhibits the virus due to the multivalent binding of the glycan receptors to the sulfates on lignin (Figure 4). Similarly, in the instance of the lignin hydrogels, the viruses were entrapped in the hydrogels due to binding with the sulfate moieties. This concept is well represented in Figure 4a. The aqueous solutions of BL, SL1, and SL2 at different concentrations were incubated with HSV-1-GFP solution (200 plaque-forming units, PFU) for 45 min at room temperature. Then, the mixture was applied to VeroE6 cells for 45 min at room temperature. After washing, the cells were infected with an overlay medium (which has carboxymethyl cellulose (CMC) and ensures plaque formation) for 48 h at 37 °C.

The virus inhibition was initially observed through live–dead fluorescence microscopy analysis. As the virus has been modified with a green fluorescent protein (GFP) gene, infected cells express GFP when they are observed under the fluorescent microscope. The uninfected cells fluoresced blue under the fluorescence microscope as they were stained by 4′,6-diamidino-2-phenylindole (DAPI). Figure 4b shows the fluorescence microscopy images of the control as well as those with the sulfated lignin at different concentrations. The cells were observed in the DAPI (blue) and GFP (green) channels, which enable quantifying the fraction of infected cells (GFP channel) across the entire cell ensemble (DAPI channel, staining the nucleus of cells). There were no plaques when the virus was pretreated with the sulfated lignin, which gives proof of the complete inhibition of the virus. It was observed that as the concentration of sulfated lignin increased from 0.05 to 5 μg/mL, the inhibition efficiency increased, which is visible through no plaque observation at 5 μg/mL. Extracting dose-dependent inhibition bar graphs from these data indicated that increasing the degree of sulfation leads to higher inhibition by approximately 6-fold (Figure 4c and Table 1). It can be observed that SL2 shows the highest inhibition followed by SL1 and BL. Here, the intrinsic antimicrobial activity of lignin (due to the presence of polyphenolic aromatic structure) can also play a certain role in the inhibitory activity of the BL. The IC_50_ values of BL, SL1, and SL2 were found to be 86.8, 2.9, and 0.5 μg/mL, respectively (summarized above in Table 1, in nM concentrations).

Further, the HSV-1 binding to lignin-based hydrogels was investigated (Figure 4d). For this, the BL-H, SL1-H, and SL2-H hydrogels were incubated with HSV-1 solutions for 1 h at 37 °C. Afterward, the number of infectious viruses in the supernatant was titrated on Vero E6 cells via a plaque assay. The virus binding with hydrogels resulted in a reduced virus titer in the supernatant. The sulfated lignin hydrogels exhibited much higher adsorption capacities than their non-sulfated counterparts. The concentration of sulfated lignin played an important role in the binding ability of the hydrogels, as the binding and inhibition of the viruses increased with an increase in the concentrations (as represented in Figure 4d). The SL1-H and SL2-H present more virus binding sites, i.e., sulfate groups, across their structures than BL-H in the case of both hydrogels and hydrogel components, i.e., lignin. It was observed that the complete adsorption of the HSV-1 occurred at a concentration of 1.5 wt % of SL1-H hydrogels and 1 wt % of SL2-H hydrogels. It can also be correlated with the increased cross-linking density and porous network of SL2-H as compared to SL1 and BL. Therefore, it can be concluded that the higher cross-linking within the hydrogels directly influences their pathogen binding ability.

Interestingly, the impact of DoS on the inhibition efficacy was much more pronounced for lignin-based hydrogels than for non-cross-linked lignin: while the addition of sulfates to BL improved the inhibitory concentration by 2 orders of magnitude (BL vs SL2, Figure 4c), a much stronger reduction of more than 4 orders of magnitude was found for the lignin-based hydrogels (BL-H vs SL2-H, Figure 4d). This indicates that hydrogel formation indeed provides an advantage over the direct application of sulfated lignin. This observation might be attributed to an optimized 3D presentation of sulfated lignin within the hydrogel and motivates further investigation on the impact of the hydrogel structure and functionalization on pathogen retention.

**Figure 5:**
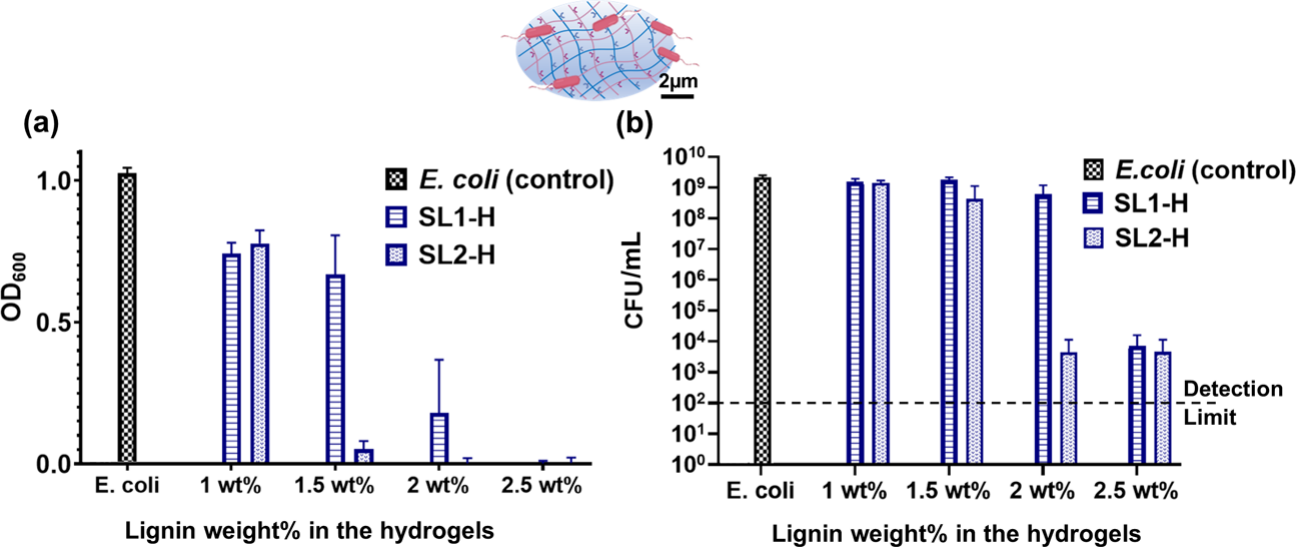
(a) Estimation of *E. coli* growth with optical density (OD) measurement of different hydrogel samples. (b) Quantitative estimation of colony-forming unit (CFU/mL) *E. coli* inhibition by lignin hydrogels.

To assess a potential broader spectrum of antimicrobial activity, we tested our compounds for activity against *E. coli* (Figure 5) and influenza A virus X31/H3N2 (Figure S12, Supporting Information). Interestingly, the sulfated lignin hydrogels showed some reduction in the virus infection for influenza A; the effect was, however, less pronounced in comparison to HSV-1 (Figure S12, Supporting Information). This inhibition effect could be attributed to the surface adsorption or adherence of the influenza A virus toward the lignin hydrogels. Upon testing the bare and sulfated lignin for their antimicrobial activity against *E. coli*, it was observed that sulfated lignins alone (i.e., before hydrogel formation) also exhibit good antimicrobial activity with complete inhibition of *E. coli* at concentrations exceeding 100 μg/mL. Interestingly, bare lignin also exhibited some antimicrobial activity at a concentration of 1000 μg/mL, which is consistent with previous published reports (Figure S13, Supporting Information).^27−29^ The developed sulfated lignin hydrogels were highly effective against *E. coli* bacteria (Figure 5), with a reduction of growth in the solution as indicated by optical density measurements (Figure 5a) and of colony-forming units by more than 5 orders of magnitude (Figure 5b). However, there was no visible effect of the bare lignin hydrogels on the *E. coli* inhibition until the concentration of 2.5 wt %, which suggests that higher concentrations are required to achieve *E. coli* inhibition from bare lignin hydrogels. The results are summarized in Figure S14 (Supporting Information).

The interaction of sulfates with the bacterium *E. coli* can be rationalized from a recent investigation of the impact of desulfation of mucins on the binding behavior of *E. coli*.^60^ It was found in this research article that mucin desulfation decreased the level of binding of *E. coli* to mucin and increased the level of attachment of the bacterium to the epithelial surface, which was attributed to the formation of interactions with surface-localized sulfated lipid and protein receptors. This indicates that sulfates are important for binding of *E. coli* bacteria to mucins and highlights the important role of sulfates as a known attachment factor for many infectious agents. Lignin is also known to generate ROS due to the presence of phenolic groups, which can play a significant role in causing oxidative stress and eventually bacterial inhibition.^61−63^

These experiments conclude that the sulfated lignin hydrogels can be applied as a sustainable alternative to inhibit a broad spectrum of pathogens and hence increase their applicability as mucus-mimicking hydrogels. These hydrogels can be potentially used as model systems for mucus research and as sustainable biomaterials for developing coatings.

## 4 Conclusions

Lignin is a promising scaffold for the large-scale synthesis of biocompatible mucus-mimicking hydrogels that exhibit antimicrobial activity. Sulfation of lignin is found to significantly improve its water solubility and interaction with sulfate-binding viruses, such as HSV-1, which increases with an increasing degree of sulfation. Tuning the concentration and degree of sulfation of the lignin in the hydrogel enabled the formation of lignin-based hydrogels that resembled native mucus with respect to the presentation of virus attachment factors and rheological properties. SEM analysis revealed that increasing the degree of sulfation significantly decreased the mesh-size distribution by orders of magnitude, which provides a novel and interesting means to fine-tune virus–hydrogel interactions. At the highest degree of sulfation (conversion of ∼90% of the available hydroxyl groups), the mesh-size distribution is on the order of native mucus (hundreds of nanometers). Interestingly, the functionalized hydrogels were also stable against enzymes (such as hyaluronidase), which are found in our body. The corresponding hydrogel shows very efficient infection inhibition, as indicated by a reduction in plaque and colony-forming units by more than 4 orders of magnitude. These experiments demonstrated that development of lignin-based hydrogels from pathogen-binding inhibitors can provide a substantial benefit in infection inhibition (attributed here to an improvement in pathogen sequestration in comparison to the non-cross-linked inhibitor). Although lignin was functionalized only with sulfates in this study, the high abundance of hydroxyl groups in lignin will enable a feasible extension toward functionalization with further pathogen attachment factors. The functionalized lignin hydrogel platform developed in this work can be used as a model system for various mucus-related disease models.

## Supporting Information Available

Materials, results, ^31^P NMR analysis, size exclusion chromatography (SEC) and elemental analysis, calculations of the maximum weight % of sulfur in each subunit of lignin, atomic force microscopy (AFM) analysis, DLS analysis of bare lignin (BL) and sulfated lignin (SL2), UV–visible spectrophotometric analysis, FTIR analysis of bare and sulfated lignin samples, rheology of hydrogels, scanning electron microscopy analysis, FTIR analysis of hydrogel in comparison with the precursors, swelling capacity: evaluation of rheological properties after reswelling of hydrogels, influenza A inhibition, and *E. coli* inhibition (PDF)
