# Supplementary material for: Lignin-Based Mucus-Mimicking Antiviral Hydrogels with Enzyme Stability and Tunable Porosity
Source: ACS Appl Mater Interfaces. 2025 Jan 29;17(6):8962–75. doi: 10.1021/acsami.4c18519 (PMC11826508; doi:10.1021/acsami.4c18519)
Supplement: Supplementary file 1 — am4c18519_si_001.pdf [file am4c18519_si_001.pdf]

Supporting Information

Lignin-Based Mucus-Mimicking Antiviral Hydrogels with Enzyme Stability and Tuneable Porosity

Sanjam Chandna[a]*, Tatyana L. Povolotsky[a], Chuanxiong Nie[a], Sophia Schwartz[a], Stefanie Wedepohl[a], Elisa Quaas[a], Kai Ludwig[a], Yulia Boyakova[a], Sumati Bhatia[b], Klas Meyer[c], Jana Falkenhagen[c], Rainer Haag[a], Stephan Block[a]*

a Institute for Chemistry and Biochemistry, Freie Universität Berlin, 14195 Germany
E-mail: sanjam1994@zedat.fu-berlin.de, stephan.block@fu-berlin.de
b Faculty of Science and Engineering, Department of Chemistry, Swansea University, Singleton Campus, Swansea SA2 8PP, Swansea, UK
c Federal Institute for Materials Research and Testing (Bundesanstalt für Materialforschung und -prüfung), Berlin, 12489 Germany

Table of Contents

1. Materials 2
2. Results 3
2.1. ^{31}P NMR Analysis 3
2.2. Size Exclusion Chromatography (SEC) and Elemental Analysis 3
2.3. Calculations of the maximum weight % of sulfur in each subunit of lignin 4
2.4 Atomic Force Microscopy (AFM) analysis 4
2.5. DLS analysis of the bare lignin(BL) and sulfated lignin (SL2) 5
2.6. UV-visible Spectrophotometric Analysis 5
2.7. FTIR analysis of the bare and sulfated lignin samples 7
2.8. Rheology of hydrogels 7
2.9. Scanning electron microscopy analysis 9
2.10. FTIR analysis of the hydrogel in comparison with the precursors 10
2.11 Swelling capacity: evaluation of rheological properties after reswelling of hydrogels 11
2.12. Influenza A inhibition 11
2.13. E. coli inhibition 11

1. Materials

Lignin, Polyacrylic acid (average M_w ~250,000, 35 wt. % in H_2O), Ammonium persulfate, Hyaluronidase (Type I-S, lyophilized powder), Sulfur trioxide pyridine complex and DAPI (4′ ,6-Diamidino-2-phenylindole dihydrochloride) were procured from Merck (Sigma Aldrich). Dulbecco's Modified Eagle Medium (DMEM), Minimum Essential Medium (MEM), Methylcellulose, Fetal Bovine Serum (FBS), LB Broth, Dulbecco's phosphate-buffered saline (DPBS), Dimethyl sulfoxide (DMSO) and Sodium Hydroxide (NaOH) were procured from Thermo Fisher Scientific. N, N-Dimethylformamide, 99.8%, Extra Dry over Molecular Sieve, AcroSeal™ was purchased from Thermo Scientific Chemicals. SnakeSkin™ Dialysis Tubing was procured from Thermo Fisher Scientific. HSV-1-GFP virus was kindly provided by Prof. Dr. Benedikt Kaufer and Prof. Dr. Klaus Osterrieder in Freie Universität Berlin. Vero E6 cells were obtained from Leibnitz Institute DSMZ – German Collection of Microorganisms and Cell Cultures GmbH.

2. Results

2.1. ^{31}P NMR Analysis

Table S1. Determination of aliphatic hydroxyl and phenolic units through ^{31}P NMR analysis (absolute integrals from NMR spectrum)

Samples	Aliphatic OH units (a.u.)	Syringyl Phenolic Units (a.u.)	Guaiacyl and Demethylated Phenolic Units (a.u.)
**BL**	31234.7	25703.1	4774.0
**SL1**	7439.5	3767.7	1612.5
**SL2**	3335.5	5002.6	1241.4

2.2. Size Exclusion Chromatography (SEC) and Elemental Analysis

(a)

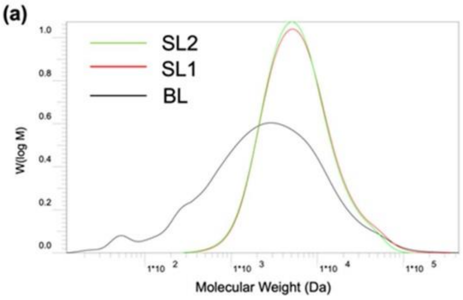

(b)

	BL	SL1	SL2
**% C**	70.90 ± 3.4	58.25 ± 1.9	50.39 ± 2.1
**% H**	4.65 ± 0.3	7.06 ± 0.3	5.04 ± 0.1
**% N**	0.76 ± 0.02	1.58 ± 0.07	0.49 ± 0.03
**% S**	4.10 ± 0.4	8.24 ± 0.6	13.89 ± 0.6

(c)

	BL-H	SL1-H	SL2-H
**% C**	72.51 ± 2.4	65.32 ± 1.85	64.86 ± 3.9
**% H**	8.72 ± 0.61	7.61 ± 0.11	7.29 ± 0.33
**% N**	0.91 ± 0.03	1.76 ± 0.05	0.62 ± 0.02
**% S**	4.98 ± 0.28	9.18 ± 0.72	14.01 ± 0.55

Figure S1. Results of (a) SEC, (b) elemental analysis of kraft and sulfated lignin and (c) elemental analysis of hydrogels

2.3. Calculations of the maximum weight % of sulfur in each subunit of lignin

The maximum weight% of sulfur was calculated in each subunit of lignin as shown below:

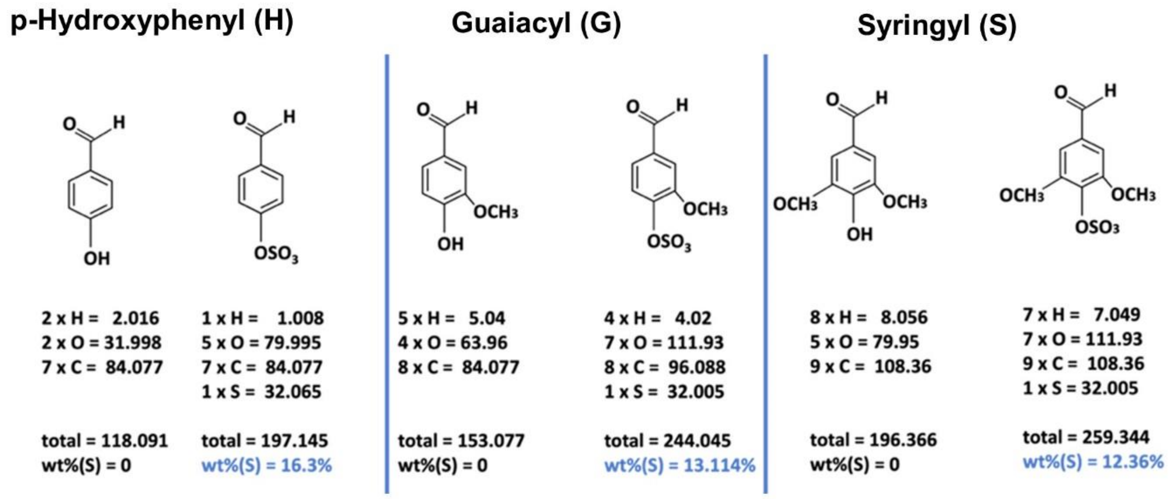

Figure S2. Calculations of the weight % of sulfur (which can be incorporated) in each subunit of lignin for an estimation of the degree of sulfation

2.4 Atomic Force Microscopy (AFM) analysis

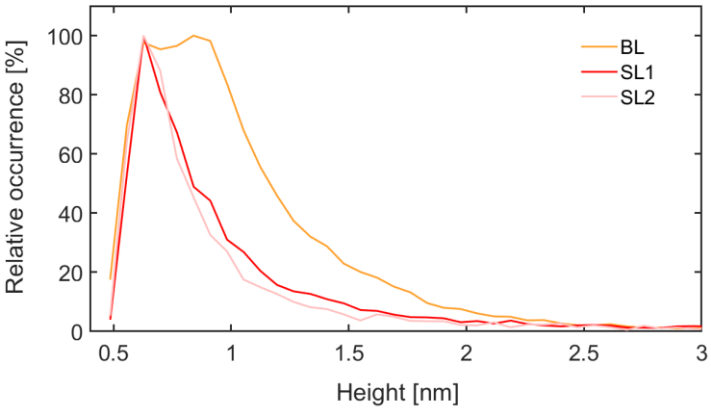

Figure S3. Particle size distribution obtained using AFM analysis

2.5. DLS analysis of the bare lignin(BL) and sulfated lignin (SL2)

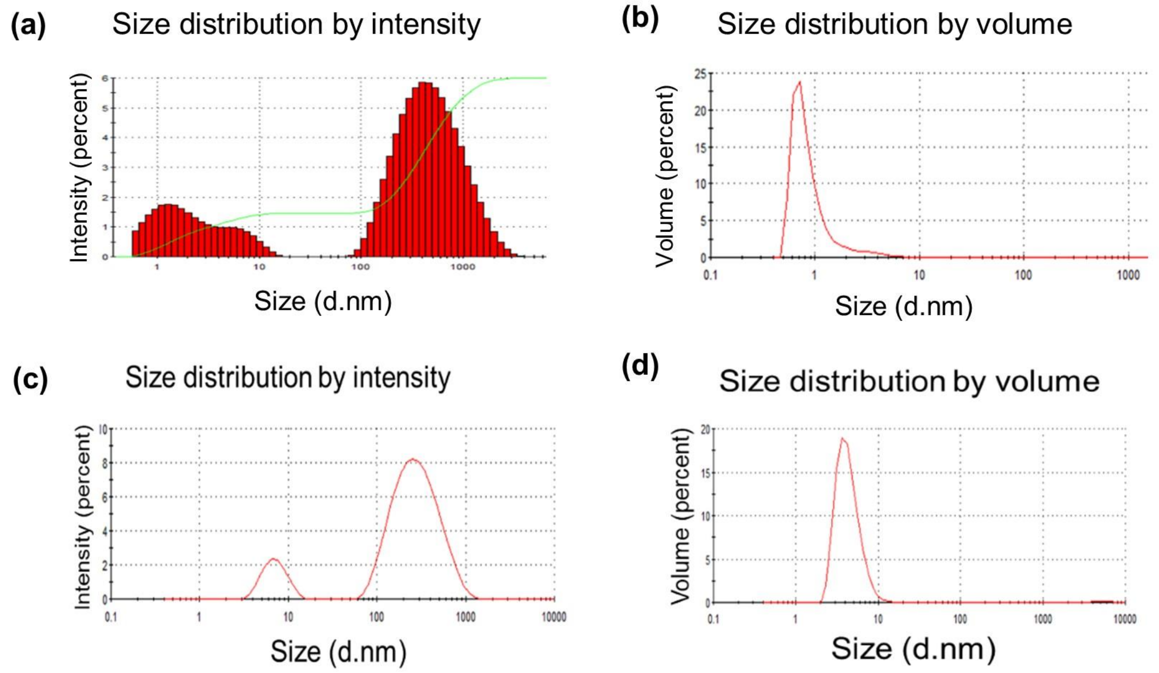

Figure S4. Particle size distribution obtained using Dynamic light scattering (DLS): (a) and (b) bare lignin (BL); (c) and (d) sulfated lignin (SL2)

2.6. UV-visible Spectrophotometric Analysis

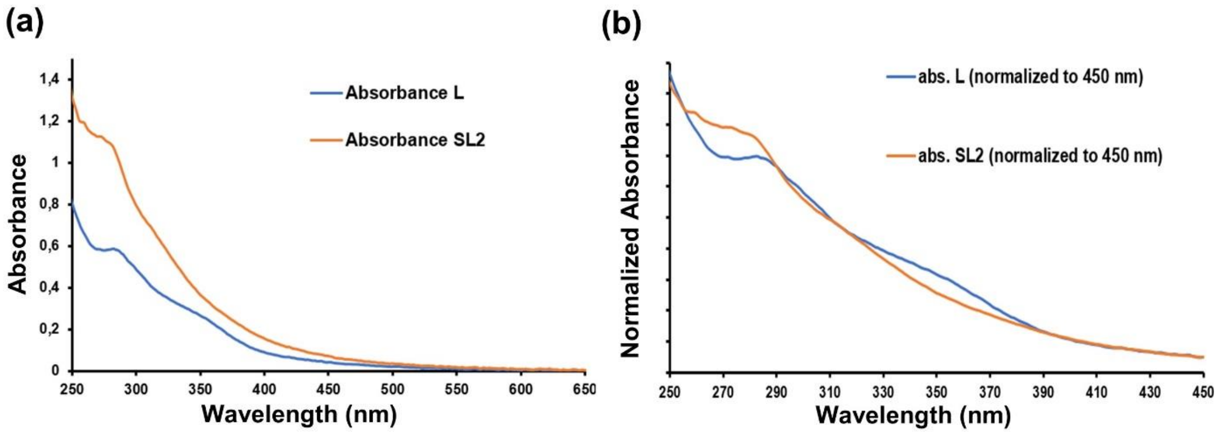

Figure S5 (i). UV-vis spectroscopic analysis of the (a) bare and (b) sulfated lignin samples.

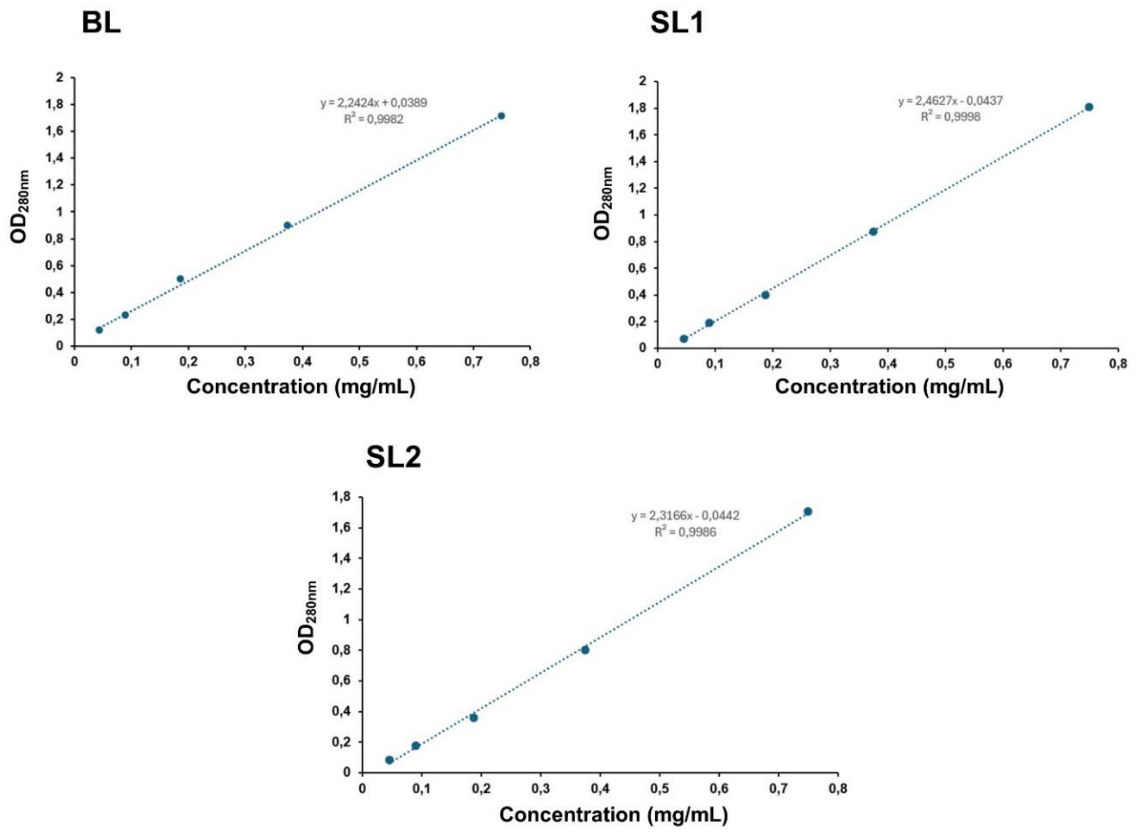

Figure S5 (ii). Standard calibration curves of bare lignin(BL) and sulfated lignin (SL1) and (SL2)

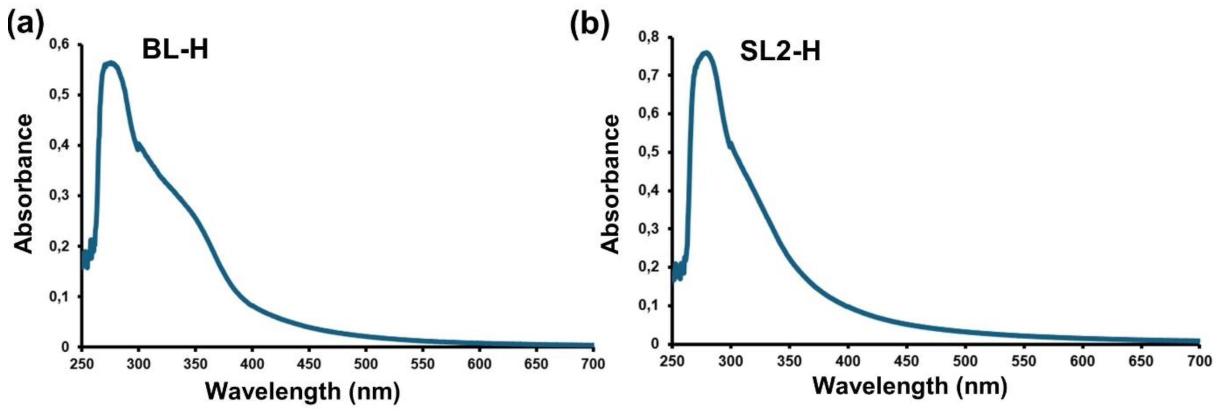

Figure S5 (iii). UV-visible spectra of lignin hydrogels showing the absorption peak at ~280 nm for both the samples (a) BL-H and (b) SL2-H.

2.7. FTIR analysis of the bare and sulfated lignin samples

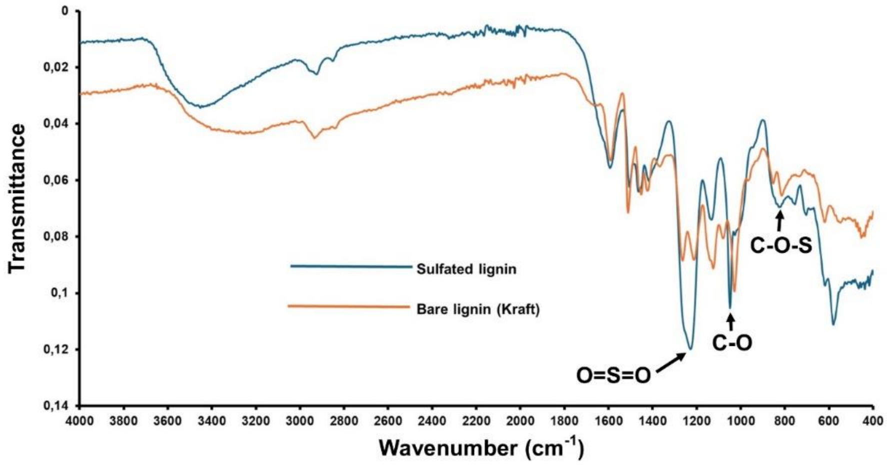

Figure S6. FTIR Analysis of the sulfated lignin in comparison to bare lignin.

2.8. Rheology of hydrogels

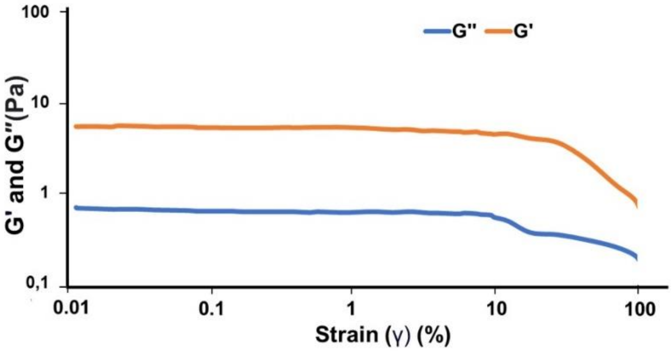

Figure S7. Strain Amplitude Sweep Tests for the hydrogels (Determination of the linear viscoelastic region through the strain amplitude sweep test at a constant Frequency of 1.0 Hz).

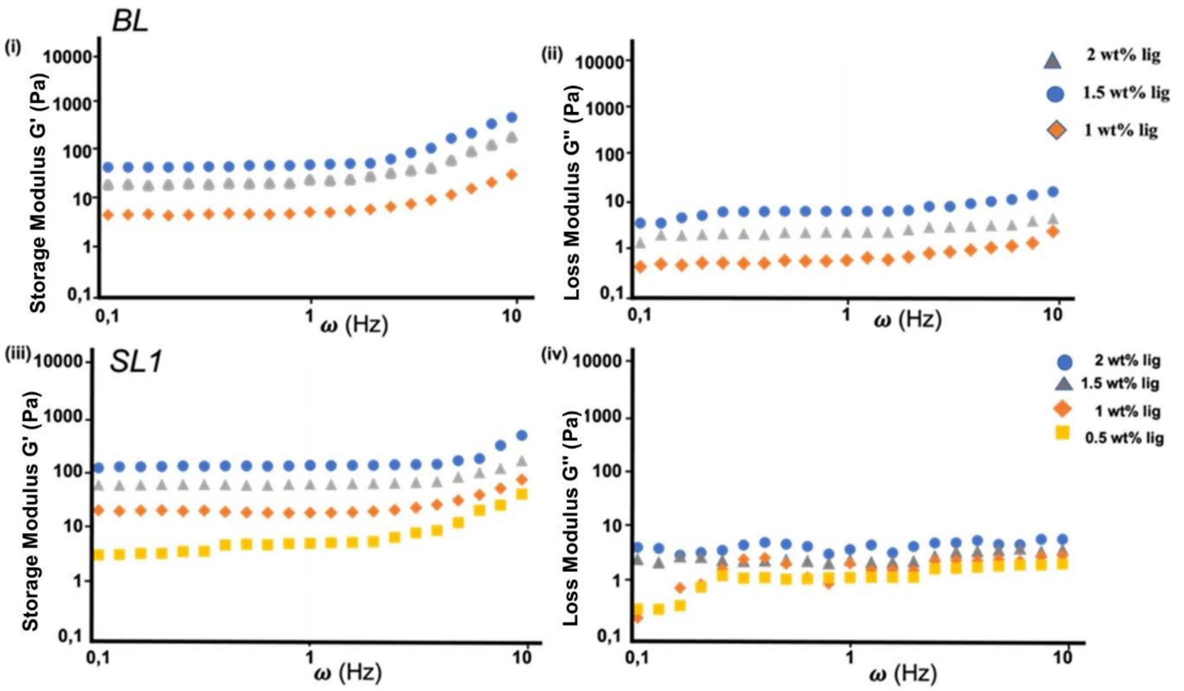

Figure S8 (a). Storage (G') and loss (G″) moduli (Pa) as a function of radial frequency (ω) of (i), (ii) bare lignin hydrogel and (iii), (iv) sulfated lignin hydrogel (SL1) in which the gel component ratios were varied systemically.

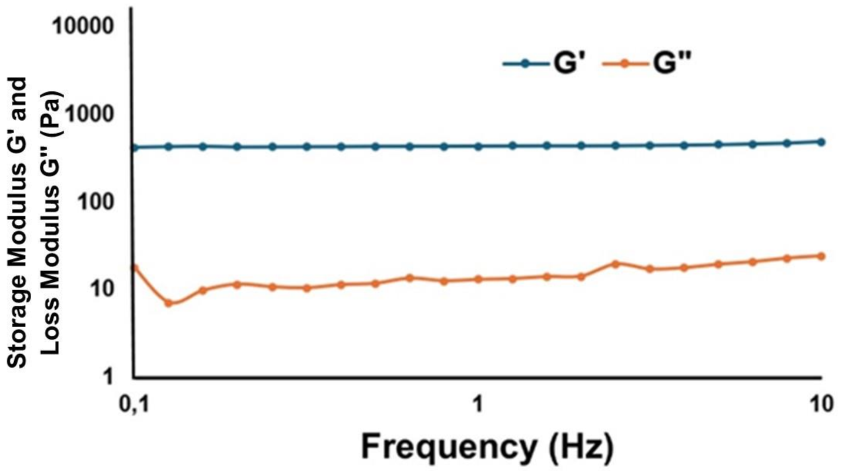

Figure S8(b). Storage (G') and loss (G″) moduli as a function of radial frequency (ω) of Polyacrylic Acid (PAA) hydrogel

2.9. Scanning electron microscopy analysis

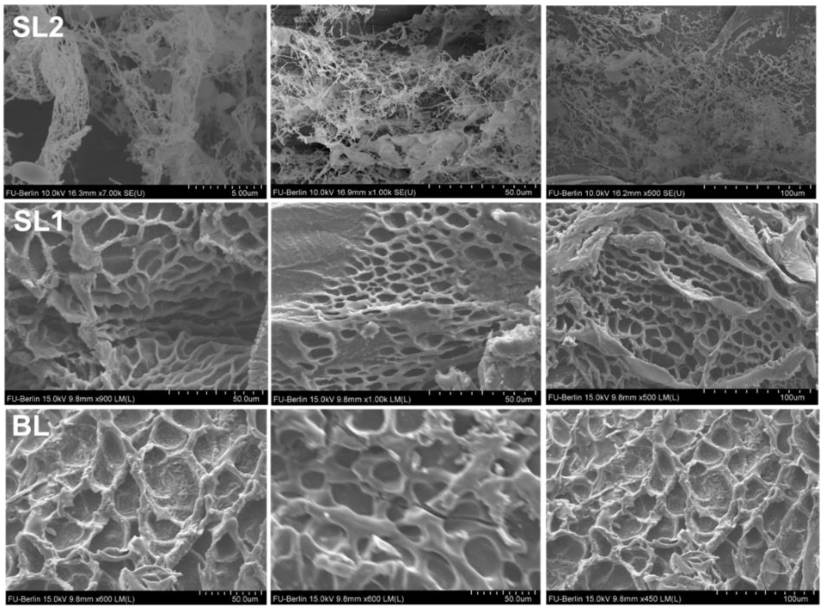

Figure S9(a). SEM Analysis of the SL2, SL1 and BL at 1 wt% concentration of lignin.

Rheological assessment of the hydrogels indicated that the sulfated lignin hydrogels exhibit an increased (shear) storage modulus G', which improves with the degree of sulfation. In the main manuscript, this behavior was attributed to a strong decrease in hydrogel mesh size, which is can be resolved in the SEM images (Figure S9) and can, apart from the reasons mentioned in the main manuscript, be attributed to be caused by:

<u>Increased Polarity and Hydrophilicity</u>: Sulfation introduces sulfate groups (-SO_3 H) to the lignin, which are highly polar and hydrophilic. This makes the lignin more soluble in water and can enhance its ability to interact with polyacrylic acid (PAA). The enhanced hydrophilicity allows better dispersion of lignin in the hydrogel matrix, leading to more uniform (finer) crosslinking.

<u>Reduced aggregation and increased particle dispersity</u>: The introduction of sulfate groups causes reduced aggregation and more dispersity of the lignin particles in the aqueous solution. These changes make crosslinking easier, as the reactive groups on the lignin are more exposed (more surface area to volume ratio) for crosslinking to occur.

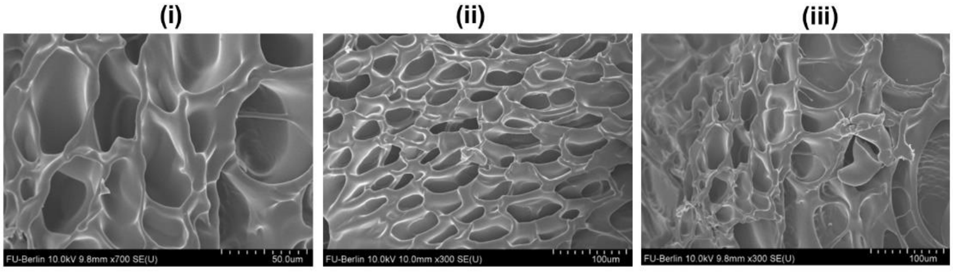

Figure S9(b). SEM Analysis of PAA hydrogels at (i) 50 µm, (ii) and (iii) 100 µm.

2.10. FTIR analysis of the hydrogel in comparison with the precursors

(a)

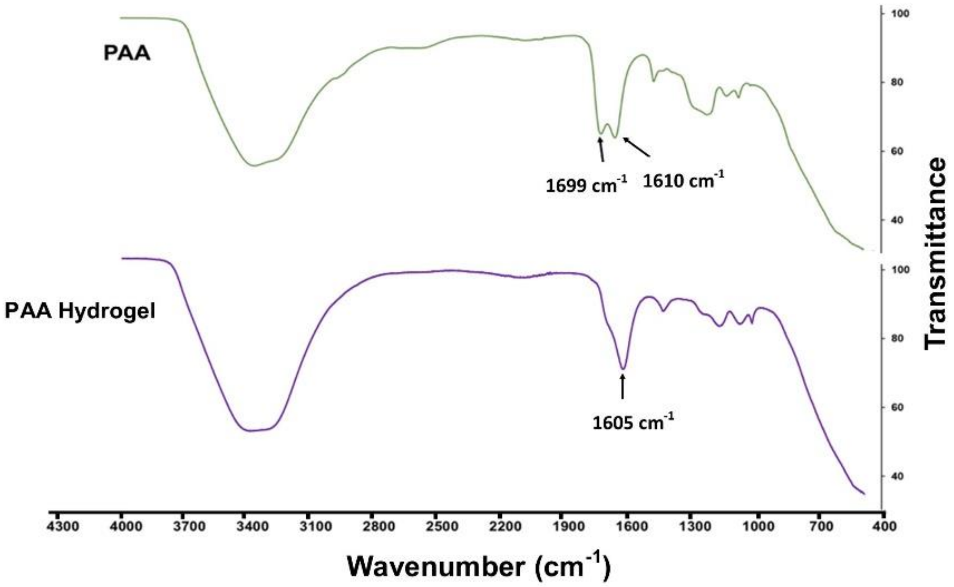

(b)

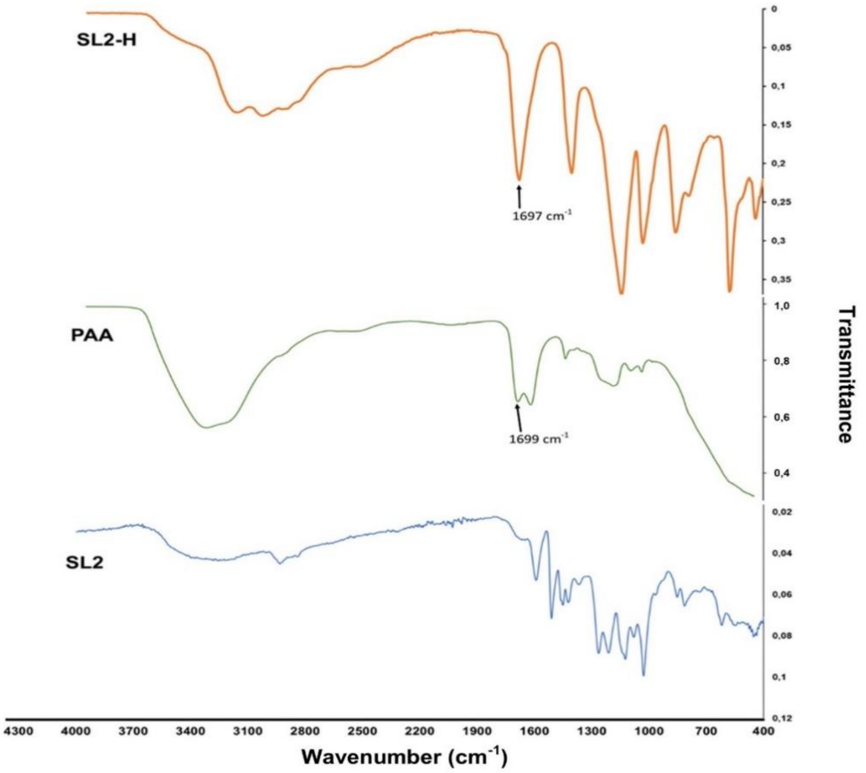

Figure S10. (a) FTIR Analysis of PAA hydrogel in comparison with PAA (b) FTIR Analysis of SL2-H (Sulfated lignin hydrogel), PAA(Polyacrylic acid) and SL2 (sulfated lignin)

2.11 Swelling capacity: evaluation of rheological properties after reswelling of hydrogels

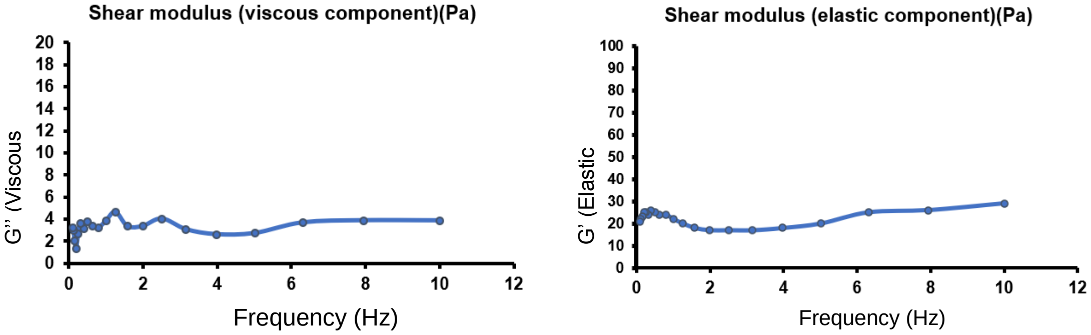

Figure S11. Evaluation of the rheological properties of the hydrogels after reswelling.

2.12. Influenza A inhibition

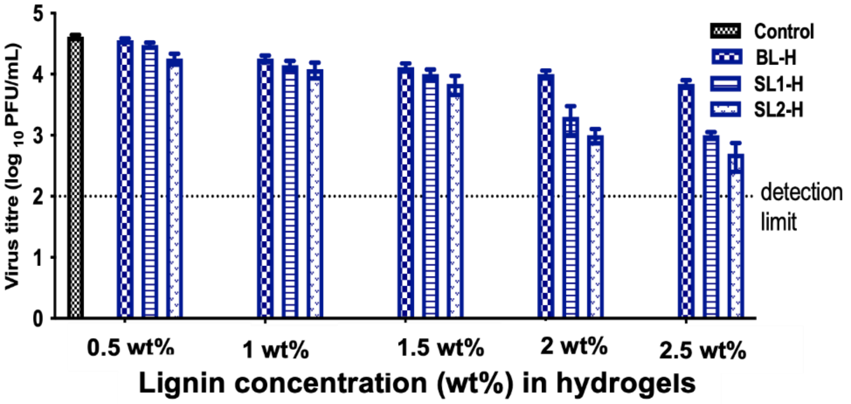

Figure S12. Quantitative estimation of Influenza A inhibition by lignin hydrogels with different weight percentages of lignin.

2.13. *E. coli* inhibition

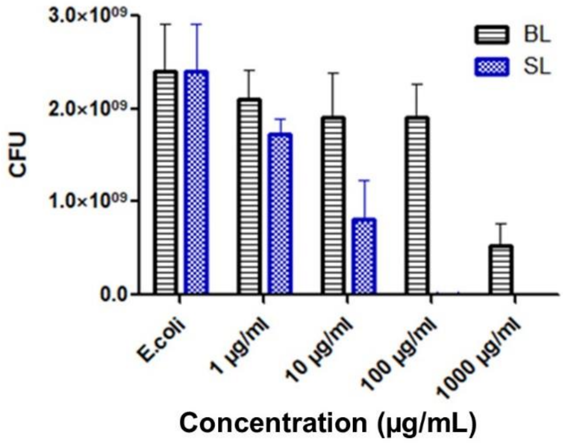

Figure S13. Effect of bare lignin and sulfated lignin (before hydrogel formation) on *E. coli* inhibition

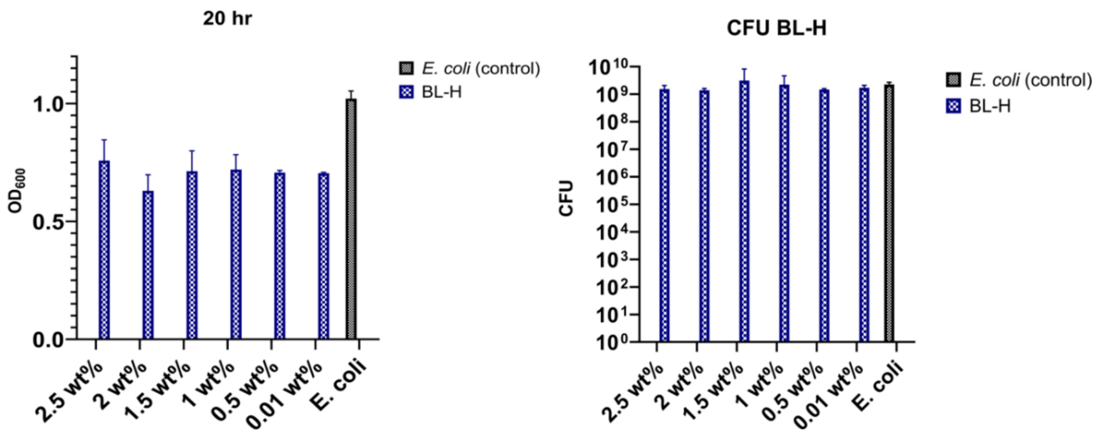

Figure S14. Effect of bare lignin hydrogels on *E. coli* inhibition
